# NanoSIMS imaging of extracellular electron transport processes during microbial iron(III) reduction

**DOI:** 10.1093/femsec/fiy104

**Published:** 2018-06-06

**Authors:** Laura Newsome, Rebeca Lopez Adams, Helen F Downie, Katie L Moore, Jonathan R Lloyd

**Affiliations:** 1 Williamson Research Centre, School of Earth and Environmental Sciences, University of Manchester, Manchester, M13 9PL, UK; 2 School of Materials, University of Manchester, Manchester, M13 9PL, UK; 3 Photon Science Institute, University of Manchester, Manchester, M13 9PL, UK

**Keywords:** electron transport, *Geobacter*, *Shewanella*, ^13^C labelling, arsenic, NanoSIMS

## Abstract

Microbial iron(III) reduction can have a profound effect on the fate of contaminants in natural and engineered environments. Different mechanisms of extracellular electron transport are used by *Geobacter* and *Shewanella* spp. to reduce insoluble Fe(III) minerals. Here we prepared a thin film of iron(III)-(oxyhydr)oxide doped with arsenic, and allowed the mineral coating to be colonised by *Geobacter sulfurreducens* or *Shewanella* ANA3 labelled with ^13^C from organic electron donors. This preserved the spatial relationship between metabolically active Fe(III)-reducing bacteria and the iron(III)-(oxyhydr)oxide that they were respiring. NanoSIMS imaging showed cells of *G. sulfurreducens* were co-located with the iron(III)-(oxyhydr)oxide surface and were significantly more ^13^C-enriched compared to cells located away from the mineral, consistent with *Geobacter* species requiring direct contact with an extracellular electron acceptor to support growth. There was no such intimate relationship between ^13^C-enriched *S*. ANA3 and the iron(III)-(oxyhydr)oxide surface, consistent with *Shewanella* species being able to reduce Fe(III) indirectly using a secreted endogenous mediator. Some differences were observed in the amount of As relative to Fe in the local environment of *G. sulfurreducens* compared to the bulk mineral, highlighting the usefulness of this type of analysis for probing interactions between microbial cells and Fe-trace metal distributions in biogeochemical experiments.

## Teaser

NanoSIMS imaging shows that metabolically-active cells of *Geobactersulfurreducens* were co-located with an iron(III)-(oxyhydr)oxide surface, consistent with this bacterium requiring direct contact with an extracellular electron acceptor.

## INTRODUCTION

The reduction of Fe(III) in minerals can have profound consequences on the mineral composition and aqueous geochemistry of the subsurface environment. One of the outcomes of the bioreduction of ferric minerals, such as ferrihydrite, is the reductive dissolution of Fe(III), which forms soluble Fe(II) and is likely to cause the release of other incorporated or sorbed elements such as arsenic (Coker *et al*. 2008; Cutting *et al*. 2009). Soluble Fe(II) can in turn recrystallise to form new Fe(II)-bearing minerals such as magnetite, siderite or vivianite depending on the pH, temperature and the other geochemical species present (Hansel *et al*. 2003). These processes can also affect the mobility of arsenic and other toxic metals and radionuclides (Lloyd *et al*. 2000; Smedley and Kinniburgh 2002; Newsome, Morris and Lloyd 2014; Watts *et al*. 2015). Focusing on the release of arsenic to groundwaters as a high profile example, this is likely caused by the development of reducing biogeochemical conditions linked to subsurface microbial activities (Islam *et al*. 2004a,b). However, As can also be re-sequestered by newly forming minerals such as vivianite and siderite (Islam *et al*. 2005; Muehe *et al*. 2016), and magnetite also was found to incorporate As(V) and create surface complexes with As(III) (Islam *et al*. 2005; Coker *et al*. 2006; Wang *et al*. 2014). Such interactions with As during the mineralisation of Fe(II)/Fe(III) minerals could potentially alter the distribution of As on the micro scale and delay its release into aquifers used to supply drinking waters on a macro scale. It is therefore crucial to understand the mechanism of microbial Fe(III) reduction in order to assess its impact on the release (and capture) of metals and metalloids. Given that the contamination of drinking water with arsenic is a major health concern that threatens the lives of more than 140 million people worldwide (Singh *et al*. 2015), this area warrants particular attention.

Fe(III) is an electron acceptor commonly used in anaerobic microbial respiration. A diverse range of bacteria and archaea covering a wide range of phylogenetic groups has been identified that enzymatically reduce Fe(III) (Lovley, Holmes and Nevin 2004). Fe(III) mostly exists in the natural environment in the solid form as oxides such as ferrihydrite, goethite and haematite, except in strongly acidic conditions where it is soluble. This means that unlike the more energetically favourable electron acceptors oxygen and nitrate, insoluble Fe(III) is unable to diffuse or be transported into the cell to be respired. As the lipid-rich cell membrane is impervious to electrons, Fe(III)-reducing microorganisms have had to evolve mechanisms for extracellular electron transfer from the surface of the cell, in order to be able to generate a proton motive force and hence conserve energy for growth. Most of the studies that underpin our knowledge of extracellular electron transport have been performed on two model organisms, the Gram-negative bacteria *Geobacter sulfurreducens* and *Shewanella oneidensis* MR1 (formerly *Alteromonas putrefaciens*), that both gain energy from reducing Fe(III) to Fe(II). These genera of bacteria were first shown to respire Fe(III) in the late 1980s (Lovley *et al*. 1987; Myers and Nealson 1988). Both *Geobacter* and *Shewanella* species use outer membrane porin-cytochrome complexes to transport electrons to the outer membrane, but the proteins involved are phylogenetically distinct and are considered to have evolved independently (Lovley, Holmes and Nevin 2004; White *et al*. 2016). They also use different mechanisms of transporting electrons from the surface of the outer membrane to the extracellular Fe(III) mineral electron acceptor, as described below.

*Geobacter* species are obligate anaerobes that couple the reduction of Fe(III) to the oxidation of acetate and other organic substrates (Lovley *et al*. 1987; Lovley and Phillips 1988). They are common in the Fe(III)-reducing zone of sediments (Lovley, Holmes and Nevin 2004), and have been found to dominate the subsurface microbial community following biostimulation of the subsurface by acetate additions during targeted uranium bioremediation programmes (Anderson *et al*. 2003; Wilkins *et al*. 2009; Kerkhof *et al*. 2011; Williams *et al*. 2011). The current consensus is that *Geobacter* species require direct physical contact with an insoluble electron acceptor in order to transfer electrons (Nevin and Lovley 2000; Lovley, Holmes and Nevin 2004), and this occurs via an electron transport chain that involves both *c*-type cytochromes and pili. *Geobacter sulfurreducens* uses multiple and parallel electron transport pathways from the inner membrane quinone pool to the OmcB cytochrome, which is partially exposed to the extracellular environment (Leang, Coppi and Lovley 2003; Malvankar *et al*. 2011; Liu *et al*. 2015; Levar *et al*. 2016). From OmcB the electrons are transported to OmcE, then to pili, and then to OmcS cytochromes that pass the electrons to the electron acceptor; without each of these cytochromes and pili, electron transport to a solid electron acceptor is compromised (Childers, Ciufo and Lovley 2002; Mehta *et al*. 2005; Reguera *et al*. 2005; Leang *et al*. 2010; Malvankar *et al*. 2011). Electron transport along the pili of *Geobacter* species has been proposed to occur via metallic-like conductivity (Malvankar *et al*. 2011; Lovley 2012; Malvankar, Tuominen and Lovley 2012; 2017). An early study did suggest that *G. sulfurreducens* could produce extracellular cytochromes (Seeliger, Cord-Ruwisch and Schink 1998), but this was later proven to be incorrect (Lloyd, Blunt-Harris and Lovley 1999; Straub and Schink 2003). More recently it appears that a pili-deficient mutant was able to restore its ability to reduce electron acceptors by releasing an extracellular *c*-type cytochrome (PgcA), but the authors note that this adaptation is likely a result of the highly controlled laboratory conditions and is unlikely to occur in the environment (Smith *et al*. 2014).

*Shewanella* species are facultative anaerobes that can couple reduction of Fe(III) to the oxidation of organics such as lactate, but not acetate (Myers and Nealson 1988; Lovley, Phillips and Lonergan 1989; Lovley, Holmes and Nevin 2004; Brutinel and Gralnick 2012). They are widespread in the environment in sediments where organic matter is being degraded and are thought to be adapted to the oxic/anoxic transition zone (Fredrickson *et al*. 2008; Edwards *et al*. 2015). It is likely that electron transport in *S. oneidensis* occurs primarily via an electron transport chain that includes flavin-cytochrome complexes on the outer membrane. Transport of electrons from the inner membrane quinone pool to the outer membrane occurs via the MtrCAB porin-cytochrome complex, the MtrC cytochrome is partially exposed at the cell surface and from there the electrons can be transferred to the extracellular electron acceptor or to the outer membrane cytochrome OmcA (White *et al*. 2016). Long-range electron transport is possible in *S. oneidensis* by the extension of the outer membrane into micrometre long nanowires (Pirbadian *et al*. 2014), along which electron transport may occur via an electron hopping mechanism (Okamoto, Hashimoto and Nakamura 2012; White *et al*. 2016), or by direct electron transfer accompanied by intermediate diffusive events (Subramanian *et al*. 2017). As well as being able to transport electrons via direct contact with an electron acceptor, *Shewanella* species can also secrete soluble extracellular electron shuttles to support an indirect reduction mechanism (Nevin and Lovley 2000; Marsili *et al*. 2008; von Canstein *et al*. 2008). Indeed, flavin-mediated electron transport has been shown to be responsible for up to 75% of electron-acceptor reduction in *S. oneidensis* (Kotloski and Gralnick 2013). The secreted flavins can also form flavo-cytochrome complexes with MtrC under anaerobic conditions (Okamoto *et al*. 2014; Edwards *et al*. 2015), and it has been argued that these bound complexes increase the rate of extracellular electron transport at the cell surface, rather than through electron shuttling via the free soluble flavin shuttles (Xu, Jangir and El-Naggar 2016). An *in vivo* study with Mn(IV)-oxides found that many *S. oneidensis* cells were planktonic and made occasional contact with the mineral surface, which could suggest electron transport via direct contact was more important than the secretion of extracellular flavins (Harris, El-Naggar and Nealson 2012). Nevertheless the role of secreted flavins in extracellular metal reduction remains controversial. Although *Shewanella* species can secrete extracellular flavins to reduce electron acceptors that were inaccessible to cells (Lies *et al*. 2005; Jiang *et al*. 2010), the likelihood of this mechanism occurring outside carefully controlled laboratory experiments has been questioned (Lovley, Holmes and Nevin 2004; Smith *et al*. 2014). Potential loss of the flavin mediator to planktonic cultures is clearly a key challenge, but the role of extracellular flavins in mediating Fe(III) and Mn(IV) reduction in biofilms remains poorly constrained.

A range of metals and metalloids can be associated with Fe(III) minerals, and one of the most intensively studied is arsenic. Like iron, arsenic is also redox active, with As(V) sorbing more strongly to Fe(III) oxyhydroxides present in aquifers than As(III) (Islam *et al*. 2004a). Arsenic can be released to solution when bacteria reduce the Fe(III) oxyhydroxides hosting the arsenic, to aqueous Fe(II), or it can be released via microbial reduction of As(V) to As(III), via dissimilatory As(V) reduction that is mediated by a periplasmic arsenate reductase encoded by the *arr* operon (Saltikov and Newman 2003; Saltikov *et al*. 2003). Although certain species of *Geobacter* and *Shewanella* are able to respire As(V), such as *G. uraniireducens* and *S*. ANA3 (Saltikov, Wildman and Newman 2005; Giloteaux *et al*. 2013), neither *G.sulfurreducens* or *S.oneidensis* MR1 are capable of catalysing this process as they do not possess the *arr* operon (Islam *et al*. 2005; Lloyd *et al*. 2011; Jiang *et al*. 2013), although both strains have been reported to have putative arsenic resistance genes (which can reduce As(V) within the cytoplasm prior to efflux of the As(III) as part of detoxification process) (Islam *et al*. 2005; Jiang *et al*. 2009; 2013; Dang *et al*. 2017).

Most previous studies have focussed on the bulk chemical effects of microbial activity, but do not provide the critical spatial information on the effect of microbial activity that is essential for determining mechanistic information. NanoSIMS is a surface based secondary ion mass spectrometry (SIMS) technique that is able to image up to seven masses and achieve a spatial resolution down to 50 nm (Herrmann *et al*. 2007b). It can be used in conjunction with stable isotope labelling (for example ^13^C and ^15^N) and is therefore particularly useful for imaging metabolically active cells that have incorporated labelled substrates. NanoSIMS, often in combination with stable isotope labelling, has been used previously to study a range of microorganisms and microbial processes, including benzene degrading communities (Schurig *et al*. 2015), periplasmic encrustation in nitrate-reducing Fe(II)-oxidising bacteria (Miot *et al*. 2015), anaerobic phototrophy (Musat *et al*. 2008), nitrogen fixation in cyanobacteria (Ploug *et al*. 2010; Woebken *et al*. 2012) and more recently syntrophic interactions between species (Wu *et al*. 2013; Gieg, Fowler and Berdugo-Clavijo 2014; Green-Saxena *et al*. 2014), as well as the location of active microorganisms in soil (Herrmann *et al*. 2007a) and the nature of organic matter in soil microenvironments (Vogel *et al*. 2014).

The aim of this study was to observe the colonisation of an As(V)-doped Fe(III)-(oxyhydr)oxide surface by different model Fe(III)-reducing bacteria. *Geobacter sulfurreducens* was selected as a model Fe(III)-reducing bacterium that requires direct contact with an extracellular electron acceptor. In contrast, *Shewanella* ANA3 was selected as a model Fe(III)-reducing bacterium that does not require direct contact with an extracellular electron acceptor. The cells were supplied with ^13^C-labelled substrates to enable the localisation of metabolically active cells in relation to the mineral surface by NanoSIMS, and therefore explore mechanisms of iron reduction. Finally, the role of microbial Fe(III) reduction (and enzymatic As(V) reduction) in controlling the fate of As(V) incorporated within Fe(III)(oxyhydr)oxide was also studied using these two organisms. As *G. sulfurreducens* is unable to reduce As(V), in this experiment the As(V) served as a tracer to allow observations of the effect of Fe(III)-reduction in the local environment of the cells. This contrasts with *Shewanella* ANA3, which is also able to reduce As(V) enzymatically.

## MATERIALS AND METHODS

### Thin film preparation

To enable visualisation of cell-mineral interfaces by microscopy with limited disturbances, supported films of Fe(III) minerals were prepared. These thin films were made using synthetic ferrihydrite, a model amorphous iron(III)-(oxyhydr)oxide mineral relevant to environmental processes and known to be bioavailable for Fe(III)-reducers. Ferrihydrite was synthesised (Schwertmann and Cornell 2000; Cornell and Schwertmann 2006) with As incorporated via the addition of Na_2_HAsO_4_ to achieve a 12% mol/mol As/Fe ratio. All reagents were analytical grade. The ferrihydrite-As suspension was added to glass slides (for optical microscopy) or boron doped silicon wafers (for SEM and NanoSIMS) by dropping a suspension onto each wafer and leaving it to dry in air.

### Cell culturing

*Geobacter sulfurreducens* was obtained from the laboratory culture collection of the University of Manchester Geomicrobiology group, and grown anaerobically in a modified freshwater enrichment medium (after Lovley and Phillips 1988) at pH 7, with 15 mM acetate as the electron donor and 40 mM fumarate as the electron acceptor. Cultures were grown in the dark at 30°C until the late-logarithmic phase, when they were harvested by centrifugation (5000*g*, 20 min), washed twice in an anaerobic 30 mM bicarbonate buffer, then suspended into the experimental medium as appropriate.

*Shewanella* ANA3 was also obtained from our laboratory culture collection and grown aerobically on LB agar plates and then inoculated into an anaerobic modified minimal medium (Saltikov *et al*. 2003) at pH 7 with 20 mM lactate as the electron donor and 40 mM sodium fumarate as the electron acceptor. Cultures were grown in the dark at 30°C until the late-logarithmic phase, when they were harvested by centrifugation (5000*g*, 20 min), washed twice in an anaerobic 30 mM bicarbonate buffer, then suspended into the experimental medium as appropriate.

### Thin film colonisation preparatory experiments

To investigate the colonisation of Fe(III) mineral coatings and associated As mobilisation by a model Fe(III)-reducing bacterium, experiments were set up comprising a glass slide coated with the iron-(oxyhydr)oxide mineral held vertically in a sterile glass serum bottle with the headspace degassed with N_2_. This was submerged in a modified anaerobic freshwater minimal medium (after Lovley *et al*. 1991) at pH 7 containing 30 mM bicarbonate, 4.3 mM phosphate and with 10 mM acetate as the electron donor and the Fe(III) thin film coating as the sole electron acceptor. Cells of *G. sulfurreducens* were added to achieve an optical density OD_600_ of 0.3. The bottles were incubated in the dark at 30°C. To assess the rate of Fe(III)-reduction and cell colonisation, samples of the medium were obtained using a degassed needle and syringe and monitored for OD_600_. At selected time points the glass slides were harvested and either analysed for cell colonisation by staining with DAPI and counting via optical microscopy (Zeiss Axio Imager A1), or for Fe(III)-reduction by dissolving in 0.5 N HCl to assess Fe(II) content via the Ferrozine assay and then 0.5 N hydroxylamine-HCl for total bioavailable Fe (Lovley and Phillips 1986).

### Thin film colonisation experiments for NanoSIMS

The methodology described above for the preparatory experiments was used to prepare samples for NanoSIMS analysis, except that conducting Si wafers were used instead of glass slides and for *G. sulfurreducens* 10 mM ^13^C-acetate (99% CH_3_^13^CO_2_Na, ISOTEC INC) or for *S*. ANA3 10 mM ^13^C-lactate (99% ^13^CH_3_CH(OH)CO_2_Na, Sigma Aldrich) were used as electron donors to label the metabolically active cells. Cells of *G. sulfurreducens* were added to an OD_600_ of 0.3 and *S*. ANA3 to an OD_600_ of 0.5. Negative controls contained no added electron donor, and controls with no bacteria (in triplicate) were used to assess the abiotic solubility of Fe and As under the experimental conditions used.

Changes in aqueous geochemistry were monitored at selected time points by removing aliquots of the medium using a degassed needle and syringe, taking care not to disturb the wafer. Aliquots were diluted in 2% HNO_3_ and monitored for Fe and As by ICP-AES (Perkin–Elmer Optima 5300 DV).

The wafers were harvested after 11 days (as this incubation time was found to be most suitable for obtaining optimal cell colonisation by DAPI staining and cell counting) and one replicate wafer was preserved for SEM and NanoSIMS analysis. The remaining aqueous phase was diluted in 2% HNO_3_ and monitored for Fe and As by ICP-AES, or diluted in deionised water and analysed for As(V) and As(III) by ion chromatography inductively coupled plasma-mass spectrometry (IC-ICP- MS) (Gault *et al*. 2005). The coatings on the remaining two wafers were dissolved in concentrated HNO_3_, and the digest was diluted in deionised water and monitored for total Fe and As content via ICP-AES in order to calculate a mass balance.

To investigate whether flavins were secreted by *S*. ANA3, the supernatants of these experiments were analysed for the presence of flavins using HPLC with a fluorescence detector (Thermo Scientific Dionex BioLC, GP50 pump, Dionex Utimate 3000 fluorescence detector set at 450 nm (excitation) and 520 nm (emission), using a C18 column with a mobile phase of 40% v/v methanol at a flow rate of 1 mL min^−1^).

### Preservation of samples for SEM and NanoSIMS

To preserve the cell structure for electron microscopy and NanoSIMS, and to maintain the stability of redox sensitive mineral phases, the wafer was removed from the experimental bottle in an anaerobic cabinet and placed immediately into an anoxic 2.5% glutaraldehyde solution in phosphate buffered saline (PBS in g/l NaCl 8, KCl 0.2, Na_2_HPO_4_ 1.42 and KH_2_PO_4_ 0.27) and left overnight (Schurig *et al*. 2013). The wafer was then placed into an anaerobic 1.5% glutaraldehyde PBS solution for 1 hour, then a 0.75% glutaraldehyde PBS solution for 1 hour, then dehydrated sequentially with 25%, 40%, 50%, 60%, 70%, 80%, 90% and 100% solutions of acetone or ethanol (Schurig *et al*. 2013), all under anoxic conditions. The wafer was dried in the anaerobic cabinet then immediately coated with gold using a sputter coater. Electron microscopy was performed after the NanoSIMS analysis using a Phillips XL30 ESEM-FEG operated in high vacuum conditions.

### NanoSIMS analysis and data processing

The samples were analysed using a Cameca NanoSIMS 50L at the University of Manchester. A 16 keV Cs^+^ primary ion beam with a current of 0.7–0.8pA was scanned over the sample surface to generate negative secondary ions. The instrument was aligned to detect ^12^C^−^, ^13^C^−^, ^16^O^−^, ^12^C^14^N^−^, ^13^C^14^N^−^, ^56^Fe^16^O^−^ and ^75^As^−^ taking care to avoid isobaric interferences. Prior to imaging, each area was implanted with a high current, defocused beam to remove the gold coating and achieve a dose of 1 × 10^17^ Cs^+^ ions cm^−2^. The areas for imaging were selected by identifying flat areas of mineral using the ion induced secondary electron image prior to imaging, in order to avoid topographical effects. A dwell time of 5000 µs/pixel was used with an aperture size of 200 µm (D1 = 3) and multiple image planes were acquired to increase the number of counts.

The OpenMIMS plugin (National Resource for Imaging Mass Spectrometry, Harvard, US) for ImageJ (Schneider, Rasband and Eliceiri 2012) was used to correct each image for dead time (44 ns) and then regions of interest (ROIs) were defined by hand using the summed ^12^C^14^N images to identify the boundary of every cell (0.41 µm^2^ ± 0.15, N = 1873). An example showing the cell ROIs for an area of the *G. sulfurreducens* sample is provided as Fig. S1 (Supporting Information). Areas of the sample that appeared particularly bright in the secondary electron images, such as along the edges of flat areas of mineral, were considered likely to have a significant contribution to the signal from topography, therefore were not included in ROIs. The cell ROIs were manually assigned to groups based on whether the cells were located on the iron–(oxyhydr)oxide mineral surface ‘cells on mineral’, next to the mineral ‘cells adjoining mineral’ or located on the wafer background where no mineral was present ‘cells on wafer’. ROIs were also defined where no cells were present on areas of iron-(oxyhydr)oxide mineral ‘mineral’ and the background of the wafer ‘wafer background’.

Average counts per pixel of ^12^C, ^13^C, ^16^O, ^12^C^14^N, ^13^C^14^N, ^56^Fe^16^O and ^75^As were extracted for each ROI for the *G. sulfurreducens* experiment, and ^12^C, ^13^C, ^16^O, ^12^C^14^N, ^28^Si, ^56^Fe^16^O and ^75^As for each ROI for the *S*. ANA3 experiment. All values were tabulated in Microsoft Excel and normalised for the total dose implanted during imaging (Table S1, Supporting Information). Normalising by the dose implanted during imaging accounts for differences in signal intensity that may result from differences in primary beam current, number of image planes and area. For *G. sulfurreducens* the ^13^C enrichment was calculated by taking a ratio of ^12^C^14^N and ^13^C^14^N to identify cells that had become enriched in ^13^C compared to natural background; the ^12^C^14^N and ^13^C^14^N signals were used as these gave higher counts and brighter images with better contrast compared to using ^13^C and ^12^C signals. As ^13^C^14^N was not measured for the *S*. ANA3 experiment, ^13^C enrichment was calculated as the ratio of ^13^C to ^12^C. The ratio of ^75^As relative to ^56^Fe^16^O was calculated to indicate where As may be relatively enriched or depleted compared to Fe. Box and whisker plots were generated to visualise the large dataset, with boxes representing the interquartile range and whiskers the 5th and 95th percentile.

It should be noted that after analysing the *G. sulfurreducens* sample, an interference from ^56^Fe^19^F^−^ was observed in the region of mass 75. Fluorine was not present in the experimental set up, and there are no known biological interactions between *G. sulfurreducens* and fluorine. However, fluorine is usually present in the analysis chamber of NanoSIMS. Although the detector was positioned on the centre of the peak of the As standard it is possible that both ^56^Fe^19^F^−^ and ^75^As^−^ contributed to the signal at this mass for the *G. sulfurreducens* sample. Given the source of the fluorine was the analysis chamber, it would be expected that the contribution from ^56^Fe^19^F^−^ would be homogeneous across the surface of the iron-(oxyhydr)oxide mineral and therefore any changes in the local environment of the cells would be caused by microbially mediated processes. Hereafter we refer to the mass 75 signal as ^75^As but we note that some of the counts are likely to have been from ^56^Fe^19^F^−^ as well as ^75^As. After the NanoSIMS analysis, wavelength dispersive scanning was performed to measure the K_a_ edges using an electron microprobe (JEOL JXA-8530F FEG-EPMA). The results showed conclusively that As was present but there was no F in the sample. The *S*. ANA sample was analysed after the identification of this interference and the detectors were aligned to avoid any issues with mass interference.

## RESULTS AND DISCUSSION

### Colonisation of Fe(III) by *Geobacter sulfurreducens*

**Figure 1.**
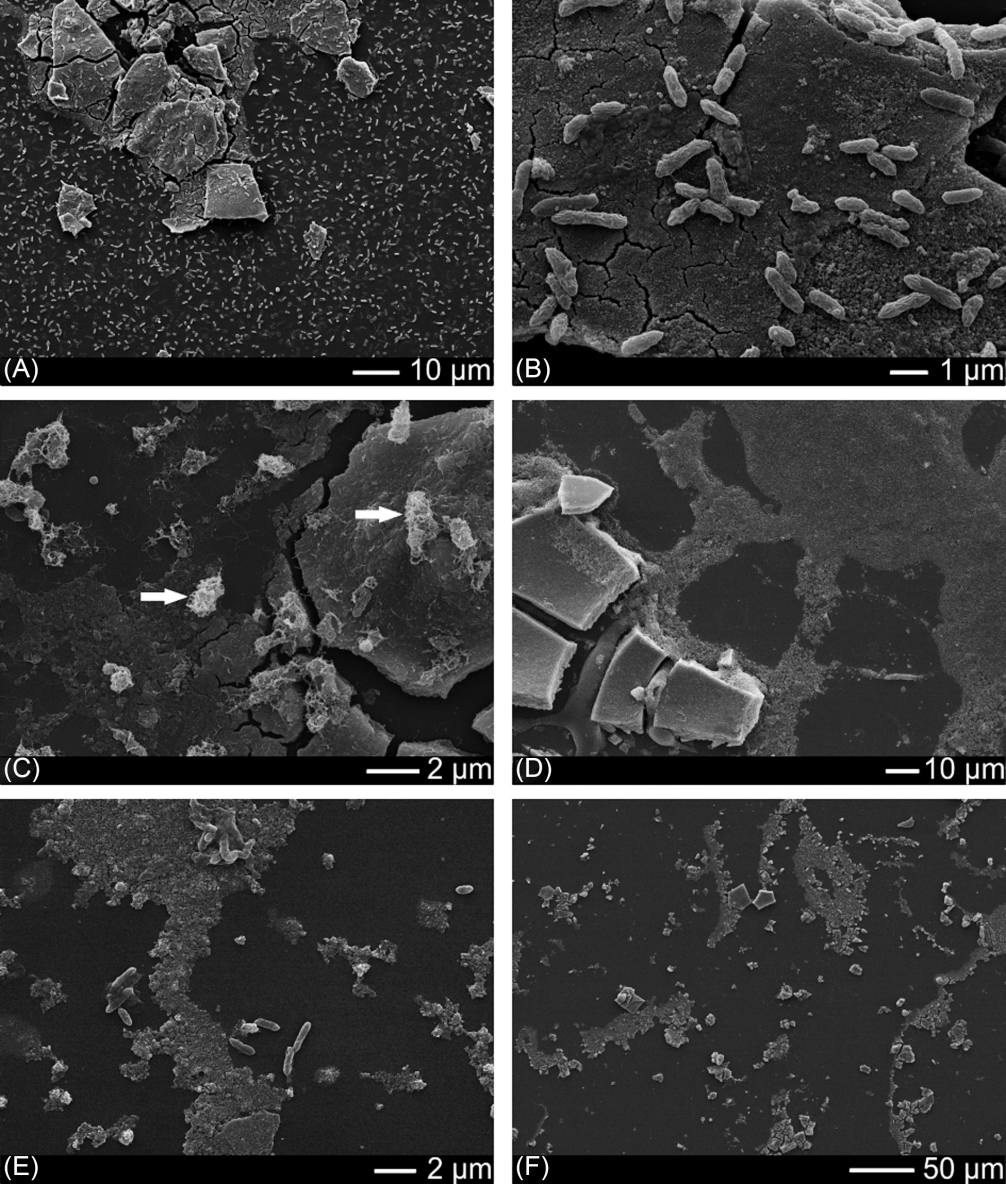
**(A-C)** SEM images showing the wafer colonised with *Geobacter sulfurreducens* after 11 days incubation with electron donor. Many cells appear to be co-located with the iron oxide coating. Arrows on Fig. 1c show example of cells coated in extracellular filamentous material. **(D-E)** Far fewer cells were present in the no electron donor controls. **(F)** The iron oxide mineral in the no cell control.

After 11 days incubation with *G.sulfurreducens* and electron donor, SEM imaging showed the wafer surface to be colonised by cells (Fig. 1a–c). Far fewer cells were visible on the wafer from the no electron donor control experiment (Fig.1d–e). The texture of the iron-(oxyhydr)oxide mineral in the no cell control was similar to that observed in the experiments with cells (Fig. 1f). Different cell morphologies were visible; some cells were present as well preserved individuals (Fig. 1a–b) while some appeared to be coated in extracellular filamentous material, which may have been involved in biofilm formation (Fig. 1c).

**Figure 2.**
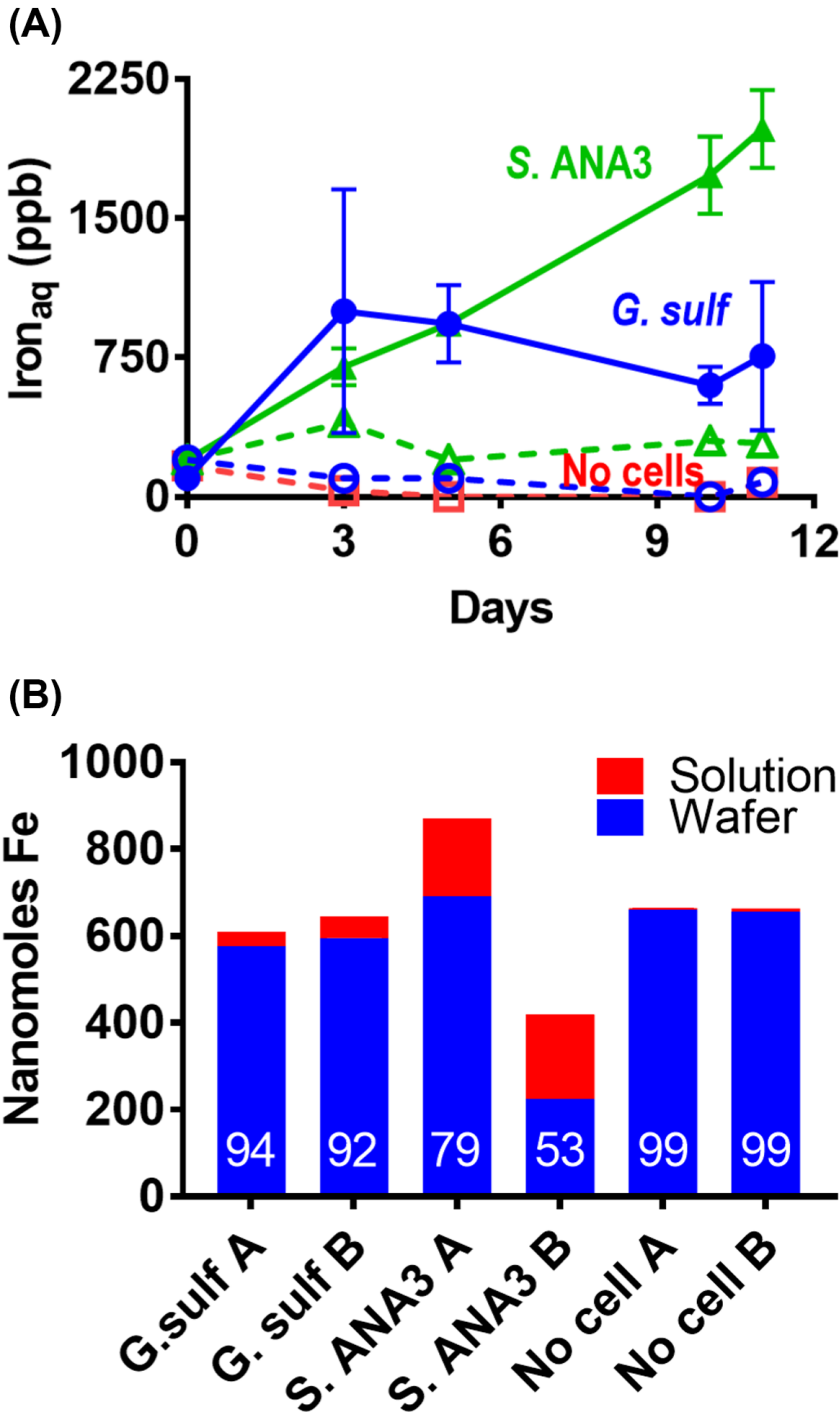
**(A)** Bulk geochemical monitoring of the aqueous phase of the NanoSIMS wafer incubations showing solubilisation of Fe in microbially active incubations. *G. sulfurreducens* is shown with circle symbols (• average 4 replicates, ○ no electron donor control), *S*. ANA3 with triangles (▴ average 3 replicates, Δ no electron donor control), and the no cell control with squares (□). Controls are shown with dotted lines and open symbols. Error bars are ±1 standard deviation. **(B)** Bulk geochemical monitoring of Fe on duplicate wafers after 11 days incubation with *G. sulfurreducens* or *S*.ANA3 supplied with electron donor compared to a no cell control. Annotations represent the % Fe on the wafer.

Geochemical measurements confirmed that soluble iron was released to solution during the 11 day experiment, presumably as Fe(II), given that Fe(III) is very poorly soluble under these experimental conditions (Fig. 2). After 11 days, up to 10% of the iron remaining in the thin film was present as Fe(II) as measured by the Ferrozine assay, after extraction with 0.5 N HCl. Minimal amounts of iron were released to solution in the controls, confirming that microbial Fe(III)-reduction was required for its release to solution.

NanoSIMS analysis was used to map the distribution of metabolically active cells in relation to the mineral substrate. Nine areas of the samples were analysed (Fig. S2, Supporting Information). Cell ROIs were assigned manually into groups based on whether the cells were located on the iron–(oxyhydr)oxide mineral surface ‘cells on mineral’ (N = 1174), next to the mineral ‘cells adjoining mineral’ (N = 117) or located on the wafer background where no mineral was present ‘cells on wafer’ (N = 582). ROIs were also defined where no cells were present on areas of iron-(oxyhydr)oxide mineral ‘mineral’ (0.25 ± 0.09 µm^2^, N = 861) and the background of the wafer ‘wafer background’ (9.55 ± 0.15 µm^2^, N = 53).

**Figure 3.**
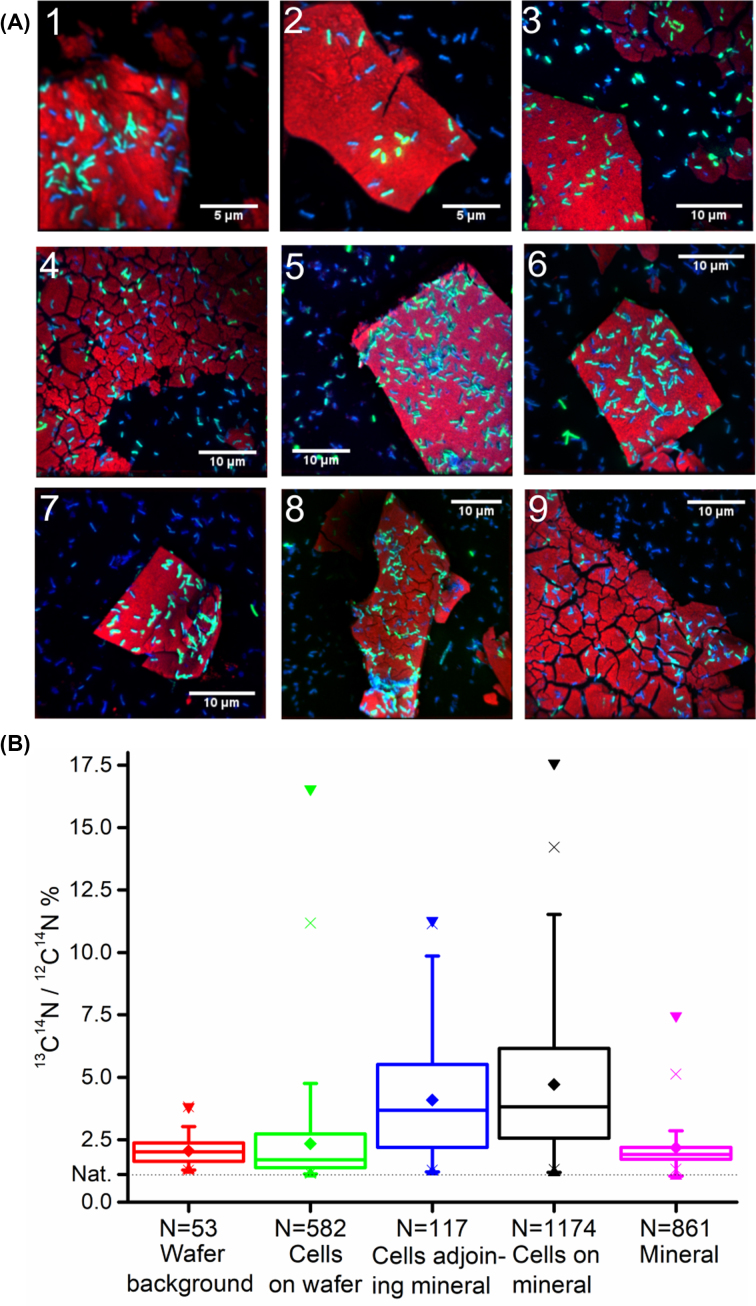
**(A)** Composite images showing the location of *G. sulfurreducens* cells in relation to mineral (^56^Fe^16^O in red, ^13^C^14^N in green, ^12^C^14^N blue). Metabolically active cells relatively enriched in ^13^C (in green) tend to be co-located with the iron oxide mineral (red), while less enriched cells tend to be located on the wafer background. **(B)** Box and whisker plots of % ^13^C calculated from ^13^C^14^N and ^12^C^14^N counts (^13^C^14^N/^12^C^14^N × 100), showing that cells located on or adjoining the iron oxide mineral were significantly more enriched in ^13^C than cells located on the wafer background (*P* < 0.001, Tukey–Kramer test). The ^13^C enrichment on the wafer background and mineral surface, were not significantly different (*P* > 0.99, Tukey–Kramer test), nor was the ^13^C enrichment of the cells on the wafer compared to the wafer background (*P* = 0.89, Tukey–Kramer test). ‘Nat’ shows known value of naturally occurring ^13^C of 1.11%.

Cells enriched in ^13^C were present in all of the areas analysed, with some enriched to more than 10 times the natural background levels. Artefacts associated with sample preparation, including dilution of ^13^C from chemical fixation (Musat *et al*. 2014) and potential interference from ^11^B^16^O^−^ meant that it was not possible to measure absolute ^13^C enrichment values. However, this was not necessary for this experiment as the ^13^C was used to locate the metabolically active cells rather than measure absolute values of ^13^C enrichment. To see where the enriched cells were located in relation to the mineral substrate, composite images were created using ImageJ for the iron-(oxyhydr)oxide mineral, ^13^C^14^N and ^12^C^14^N. These clearly showed that overall, the cells that were metabolically active and relatively enriched in ^13^C tended to be co-located with the iron-(oxyhydr)oxide mineral, whereas the cells that were less enriched in ^13^C tended to be located on the wafer background (Fig. 3a). There were some exceptions to this, such as in Area 6 where there was a cluster of cells that were highly enriched in ^13^C to the left of the iron–(oxyhydr)oxide mineral, which may have been redistributed during fixation.

Box and whisker plots were made of all cell ROIs to further display the distribution of ^13^C enrichment (Fig. 3b). The results showed that cells located on or adjoining the iron-(oxyhydr)-oxide mineral were significantly more enriched in ^13^C (*P* < 0.001) and therefore metabolically active, compared to cells located on the wafer background away from the iron-(oxyhydr)oxide mineral. The ^13^C enrichment values for the wafer background and the mineral surface were slightly higher than natural background; this is likely due to sorption of some of the ^13^C-acetate. Cells on the wafer were not significantly different in ^13^C enrichment compared to the wafer background (*P* = 0.89).

### Colonisation of Fe(III) by *Shewanella* ANA3

**Figure 4.**
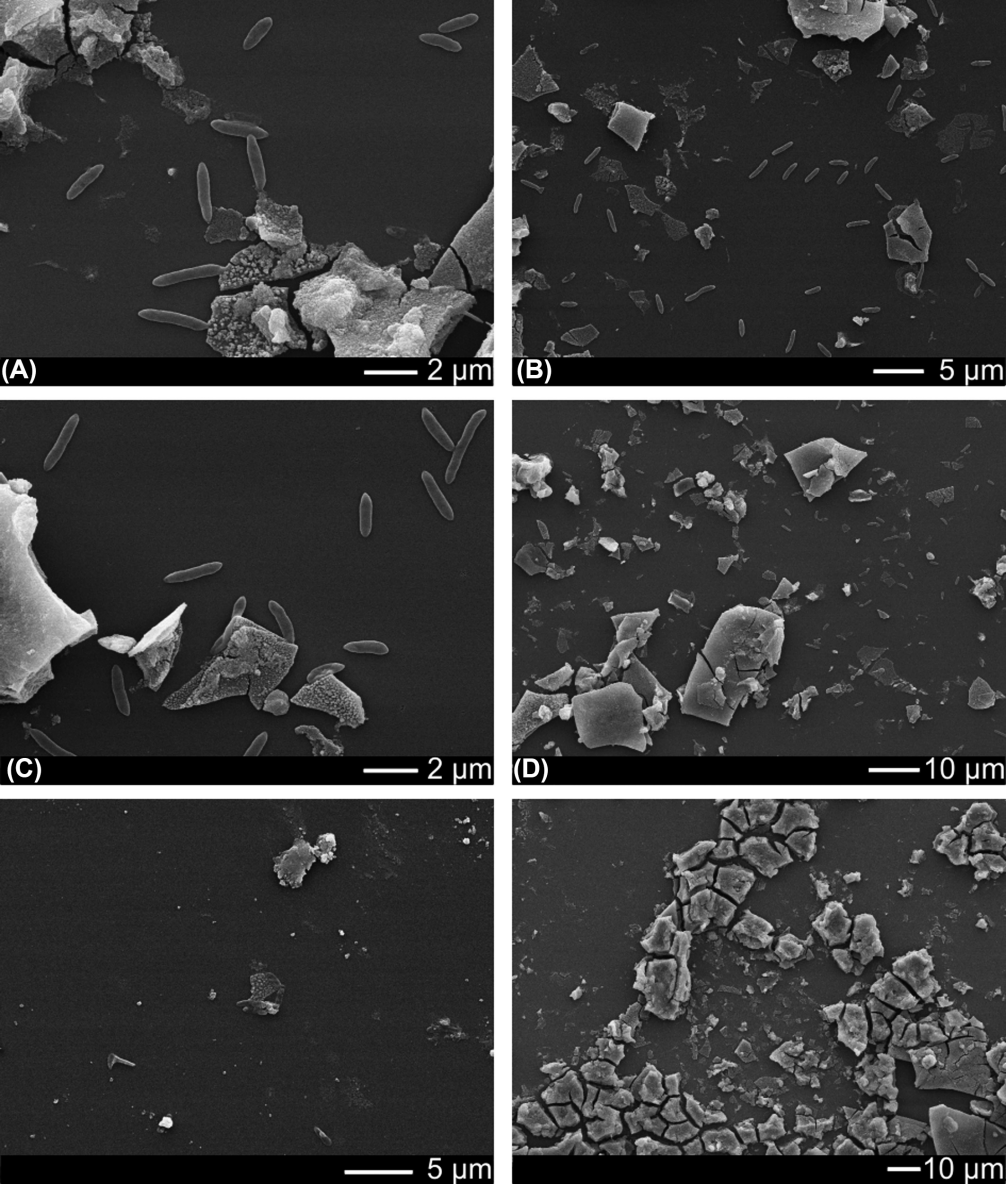
**(A-D)**SEM images showing the wafer colonised with *Shewanella* ANA3 after 11 days incubation with electron donor. Cells are located next to the iron oxide mineral and also on the uncoated wafer surface. **(E-F)** Almost no cells were visible present in the no electron donor controls.

After 11 days incubation, SEM imaging confirmed that the wafer surface was colonised by cells (Fig. 4a–d); some were in contact with (adjoining) the iron-(oxyhydr)oxide coating while others were located on the wafer background. It is noteworthy that unlike with *G. sulfurreducens*, almost no *S*. ANA3 cells were located directly on the iron-(oxyhydr)oxide mineral surface. Very few cells could be observed in the no electron donor control experiment (Fig. 4e–f). The texture of the iron-(oxyhydr)oxide mineral in the no cell control was similar to that observed in the experiments with cells (Fig. 1f). Again, geochemical measurements confirmed Fe(III) reduction occurred during the 11 day experiment (Fig. 2a). A greater quantity of Fe was released to solution with *S*. ANA3 compared to *G. sulfurreducens* (Fig. 2b), perhaps due to the higher cell density of the *S*. ANA3 experiment.

Again, NanoSIMS analysis was used to locate metabolically active cells in relation to the mineral. Three areas of the sample were analysed (Fig. S3, Supporting Information). Cell ROIs were manually assigned to the groups, ‘cells adjoining mineral’ (N = 11), and ‘cells on wafer’ (N = 43). No cells were visible on the mineral surface therefore there were no ROIs for this group. ROIs were also defined for ‘mineral’ (1.92 ± 0.96 µm^2^, N = 31) and the ‘wafer background’ (3.35 ± 3.17 µm^2^, N = 32).

**Figure 5.**
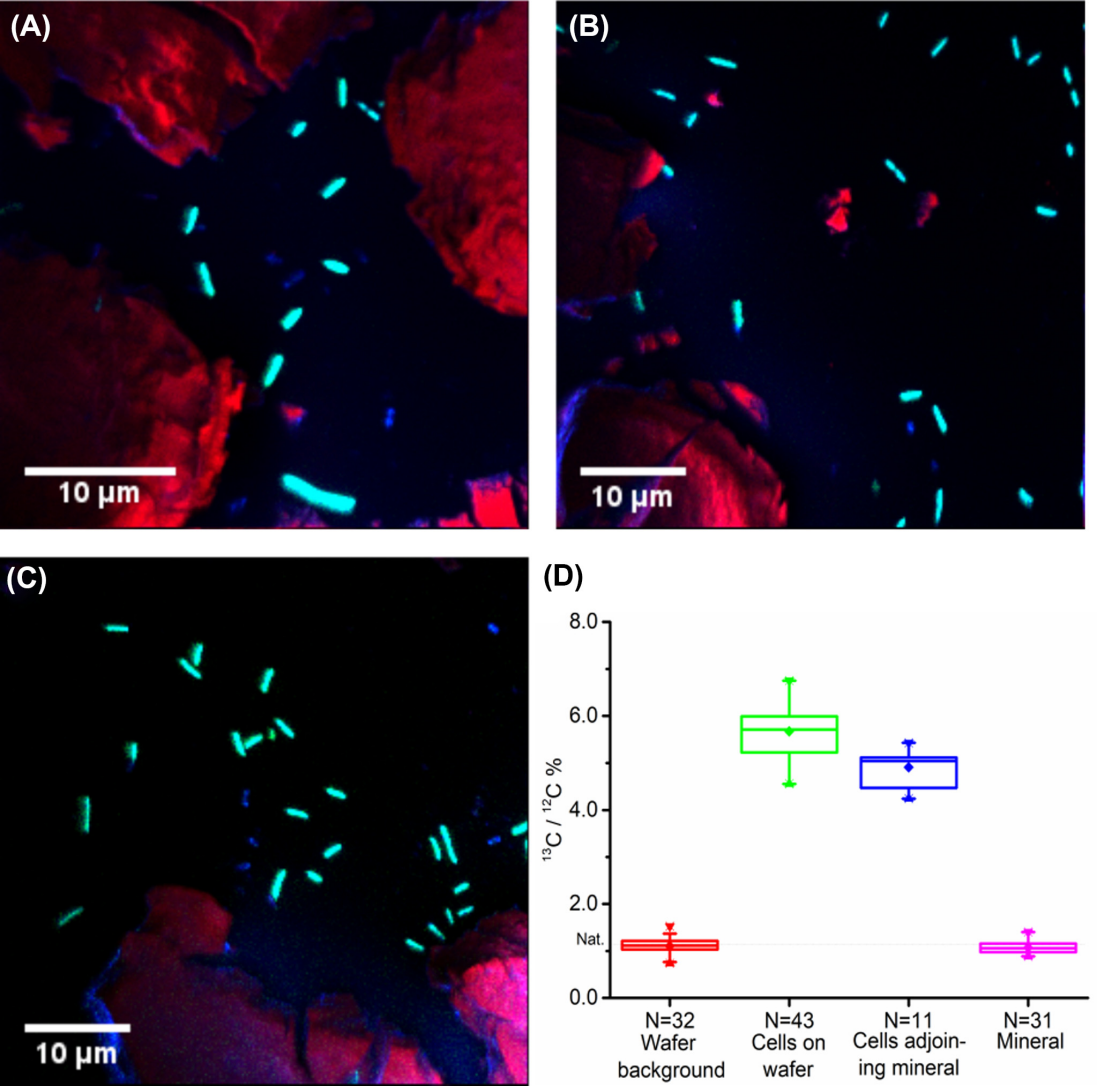
**(A-C)** Composite images showing the location of *S*. ANA3 cells in relation to mineral (^56^Fe^16^O in red, ^13^C in green, ^12^C^14^N blue). No metabolically active cells relatively enriched in ^13^C (in green) were co-located with the iron oxide mineral (red). **(D)** Box and whisker plots of % ^13^C calculated from ^13^C and ^12^C counts (^13^C/^12^C × 100), showing that cells located on the wafer background or adjoining the iron oxide mineral were significantly more enriched in ^13^C than the wafer background and mineral surface (*P* < 0.001 for all combinations, Tukey–Kramer test). The ^13^C enrichment on the wafer background and mineral surface were not significantly different (*P* > 0.98, Tukey–Kramer test). ‘Nat’ shows known value of naturally occurring ^13^C of 1.11%.

Cells enriched in ^13^C were present in all of the areas analysed (Fig. 5a–c), with some cells enriched up to seven times the natural background levels (Fig. 5d). The lack of cells directly located on the mineral surface suggests that the metabolic activity of *S*. ANA3 was not correlated with the presence of the iron-(oxyhydr)oxide mineral. The *S*. ANA3 cells located on the wafer background were highly enriched in ^13^C, in stark contrast with the *G. sulfurreducens* cultures, where the equivalent cells were not significantly enriched with ^13^C compared with the natural background. This clearly demonstrates the different mechanisms of electron transport to extracellular Fe(III) minerals used to support the metabolism of these two bacteria.

### Use of NanoSIMS to infer mechanisms of extracellular electron transport

Our experimental set up was successful in supporting the growth of cells of *G. sulfurreducens* on a thin film of iron(III)-(oxyhydr)oxide. Cells located on or adjoining the mineral were significantly more enriched in ^13^C and therefore metabolically active compared to those located on the wafer surface. This is consistent with *G. sulfurreducens* requiring direct contact with an extracellular electron acceptor (i.e. the Fe(III)-(oxyhydr)oxide mineral surface) to transfer the electrons required for respiration, via outer membrane cytochromes or conductive pili extending from the cells surface (Lovley 2012). We believe this to be the first time that this relationship has been visualised using ^13^C labelling on an *in situ* sample.

In contrast, there were almost no cells of *S*. ANA3 located on the iron-(oxyhydr)oxide mineral surface, and the cells adjoining the mineral or on the wafer background were similarly enriched in ^13^C. This is consistent with previous results showing that *Shewanella* species do not require direct contact with an extracellular electron acceptor for electron transport to occur (Lies *et al*. 2005). Instead, electron transport may occur via flavo-cytochrome complexes on the outer surface of the cells, which make intermittent contact with the mineral surfaces via chemotaxis (Harris *et al*. 2010; Harris, El-Naggar and Nealson 2012) or alternatively by secretion of extracellular flavins that acted as an electron shuttle (von Canstein *et al*. 2008). Compared to the no cell control where riboflavin was not detected, when *S*. ANA3 cells were present riboflavin concentrations between 60 and 91 nM were measured, similar to those previously observed for *S. oneidensis* MR1 (von Canstein *et al*. 2008), although unlike in this previous study no extracellular flavin mononucleotide was detected in this experiment. This suggests that the secretion of extracellular flavins by *S*. ANA3 may have contributed to electron transport in this system.

### Impact of microbial Fe(III)-reduction on arsenic solubility and speciation

**Figure 6.**
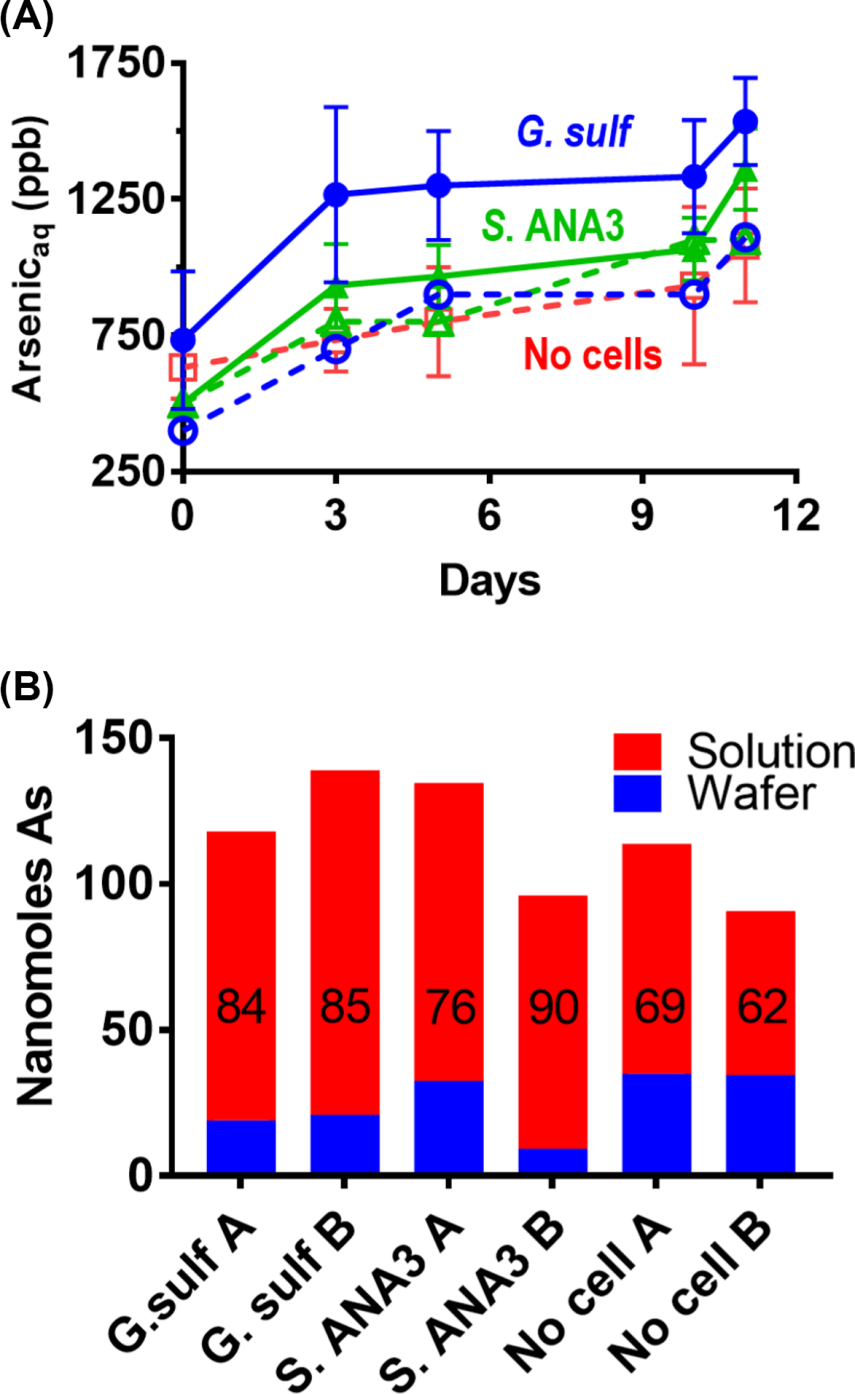
**(A)** Bulk geochemical monitoring of the aqueous phase of the NanoSIMS wafer incubations showing solubilisation of arsenic. At the end of the experiment there was more As in solution in the microbially active incubations. After subtracting the no cell control data there was 30 ± 18 nmol of As in solution after incubation with *G. sulfurreducens* and 19 ± 17 nmol of As after incubation with *S*. ANA3. *G. sulfurreducens* is shown with circle symbols (• average 4 replicates and ○ no electron donor control), *S*. ANA3 with triangles (▴ average 3 replicates and Δ no electron donor control), and the no cell control with squares (□). Controls are shown with dotted lines and open symbols. Error bars are ± 1 standard deviation. **(B)** Bulk geochemical monitoring of As on duplicate wafers after 11 days incubation with *G. sulfurreducens* or *S*.ANA3 supplied with electron donor compared to a no cell control. Annotations represent the % As in solution.

To further explore the impact of extracellular electron transfer on redox-active trace elements associated with Fe(III) minerals during bioreduction, the fate of the incorporated As(V) was determined. After 11 days of incubation, As was released to solution (Fig. 6a). Some arsenic was solubilised as As(V) in the no electron donor controls and the no cell control, which indicates that As was partially soluble under these experimental conditions. This may have been due to the experimental set up that comprised a relatively large volume of minimal medium to a small quantity of mineral, with a large surface area presented as a thin film. Slightly more arsenic was released to solution in the microbially active incubations suggesting that microbial Fe(III)-reduction had somewhat enhanced the release of arsenic under these experimental conditions.

Speciation of aqueous As (IC-ICP-MS) showed that 99.9% was present as As(V) with *G. sulfurreducens*, and 21% As(V) and 79% As(III) (± 1.6%) with *S*. ANA3, as expected given that *G. sulfurreducens* lacks the operon that encodes for respiratory As(V) reduction. It is noteworthy that after incubation with the model As(V) reducer *S*. ANA3, the majority of As had been reduced to As(III), which suggests that at least some of the aqueous-phase As(V) that was released from the mineral abiotically must have been respired, as well as the As(V) that was likely released during microbial Fe(III)-reduction. Previous work has also noted the co-dependency of the rate of As(V) release from ferrihydrite and the rate of formation of As(III) by *Shewanella* species (Revesz, Fortin and Paktunc 2016).

The amount of iron and arsenic present on the wafers after 11 days was measured and compared to the amount that was solubilised to assess whether arsenic had been preferentially released from the mineral matrix to solution compared to Fe(II). Analyses were performed on the two replicate wafers that were not preserved for SEM and NanoSIMS, and the data were calculated as nanomoles of Fe or As per experiment (Fig. 6b). These bulk measurements were used to estimate that post-incubation with *G. sulfurreducens*, there was 3.4 ± 0.1 nmol As per 100 nmol Fe present on the wafer, compared to 4.4 ± 0.4 nmol As per 100 nmol Fe with *S*. ANA3 and 5.3 ± 0.03 nmol As per 100 nmol Fe in the no cell control. All values were substantially lower than the 12 mol/mol starting value, showing that more than 50% of the As (relative to Fe) in the thin films was solubilised abiotically under these experimental conditions, also reflected in the aqueous geochemistry results (Fig. 6a). The values for the *G. sulfurreducens* wafers were significantly different to the no cell controls at the 95 % confidence level, confirming that the presence of metabolising *G. sulfurreducens* cells led to more As being released to solution relative to Fe, over and above the abiotic As solubilisation. The values for the *S*. ANA3 wafers were not significantly different to the no cell control wafers or the *G. sulfurreducens* wafers at the 95 % confidence level, but this was affected by the higher variation in the duplicate *S*. ANA3 wafer measurements, suggesting more replicates would be required to conclusively show whether or not the presence of *S*. ANA3 caused increased mobilisation of As from the wafer relative to Fe.

### Using the relationship between As and Fe to observe the effect of microbial Fe(III)-reduction by *Geobacter sulfurreducens*

Data were extracted from the NanoSIMS analysis (Fig. S2, Supporting Information) to assess whether the distribution of iron and arsenic was different in the local environment of the cells, and whether this was different to the bulk geochemical measurements. Given that *S*. ANA3 was not co-located with the mineral surface, the *G. sulfurreducens* data set was used for this analysis, which aimed to determine if NanoSIMS could be used to probe the direct impact of microbial metabolism on trace metals associated with Fe(III)-(oxyhydr)oxides.

**Figure 7.**
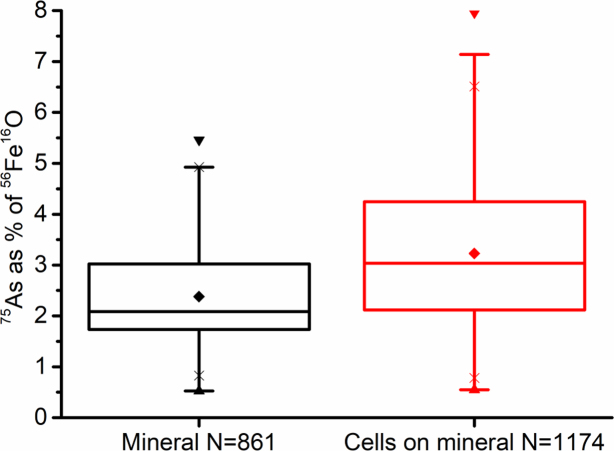
Box and whisker plot of ^75^As counts as a percentage of ^56^Fe^16^O counts illustrated for the entire *G. sulfurreducens* dataset. The proportion of ^75^As relative to ^56^Fe^16^O was significantly different in the local environment of the cells compared to the un-colonised mineral surface (*P* < 0.001, one way ANOVA). Similar results were observed when each individual area of the sample was analysed separately, see Figure S4(Supporting Information).

The ratio of ^75^As to ^56^Fe^16^O was calculated for each individual cell ROI and for the ROIs on the mineral surface where no cells were present to assess the impact of the cells on the As/Fe distribution of the mineral (Fig. 7). The box and whisker plots clearly demonstrate that the distribution of ^75^As relative to ^56^Fe^16^O was significantly different in the local environment of the *G. sulfurreducens* cells compared to the mineral surface where no cells were present. There was some heterogeneity between individual areas of the sample (Fig. S4, Supporting Information), highlighting the importance of making multiple measurements even within the same sample.

To hypothesise the processes that could lead to differences in the relative amount of As in the local environment of *G. sulfurreducens*, we reason that the inoculated cells would first become associated with the wafer surface through diffusion, electrostatic attraction or chemotaxis. The cells that had direct contact with the iron(III)-(oxyhydr)oxide mineral would then begin to respire the Fe(III) coupled to the use of ^13^C-acetate. This would lead to the release of aqueous Fe(II) and the consequent release of As(V) to solution, over and above what occurred in the no cell and no electron donor controls, probably due to the microbially-induced dissolution of the Fe(III) mineral via reduction and solubilisation as Fe(II). Here, in the local environment of the cells, there was proportionally more As present compared to where no cells were located, showing the preferential release of Fe(II) from the mineral, compared to As(V). Although the ^75^As relative to ^56^Fe^16^O results from the NanoSIMS analysis were supported by similar measurements from bulk geochemical monitoring, care should be exercised when interpreting these findings as the ionisation yields for ^75^As^−^ and ^56^Fe^16^O^−^ are not the same and will be affected by matrix effects.

### Use of NanoSIMS to assess the impact of microbial metabolism on bulk and trace elements

Using NanoSIMS was an effective way to visualise the relationship between active Fe(III)-reducing bacteria and a solid Fe(III) electron acceptor substrate. The contrasting results for bacteria that use different mechanisms of extracellular electron transport illustrates the requirement for direct contact with the substrate and consequent mineral attachment with *Geobacter* sp., compared to the ability of *Shewanella* sp. to conduct extracellular electron transport without attaching to the mineral surface. This technique could be useful to study a wide range of Fe(III)-reducing microorganisms, alongside biochemical and genetic analyses to fully understand the mechanisms of electron flow to Fe(III) and other insoluble electron acceptors.

Given that the environmental behaviour of many trace elements is controlled by Fe/Mn biogeochemistry, using NanoSIMS could provide new insights into how their fate could be controlled by microbial processes. Our findings using arsenic as an example of a trace element show that there appears to be differences in the proportion of arsenic relative to iron in the local environment surrounding metabolically active cells, compared to bulk geochemical measurements. Further work needs to be done to investigate this effect. It is likely that *S*. ANA3 reduced the bulk of As(V) to As(III) in the aqueous phase, and again additional work will be required to confirm this. In aquifer systems, the ability to respire As(V) that is mobilised during the reductive dissolution of Fe(III)-(oxyhydr)oxides, will result in the formation of poorly sorbing As(III), which will accumulate in groundwaters once available sorption sites in the aquifer have been saturated (Gnanaprakasam *et al*. 2017). This work opens the possibility for more detailed studies on Fe-As coupling using model systems and mixed community studies, using synthetic minerals and also aquifer materials. This type of analysis could be highly useful for probing Fe-trace metal distributions when studying a broader range of coupled processes, for example, Mn/Fe/S systems, and their impact on biogeochemical cycling of toxic metals and radionuclides such as Se, I and U.

## Supplementary Material

Supplementary Data

## References

[bib1] Anderson RT, Vrionis HA, Ortiz-Bernad I Stimulating the *in situ* activity of *Geobacter* species to remove uranium from the groundwater of a uranium-contaminated aquifer. Appl Environ Microbiol. 2003;69:5884–91. 14532040 10.1128/AEM.69.10.5884-5891.2003 PMC201226

[bib2] Brutinel ED, Gralnick JA Shuttling happens: soluble flavin mediators of extracellular electron transfer in *Shewanella*. Appl Microbiol Biotechnol. 2012;93:41–8. 22072194 10.1007/s00253-011-3653-0

[bib3] von Canstein H, Ogawa J, Shimizu S Secretion of flavins by *Shewanella* species and their role in extracellular electron transfer. Appl Environ Microbiol. 2008;74:615–23. 18065612 10.1128/AEM.01387-07 PMC2227709

[bib4] Childers SE, Ciufo S, Lovley DR *Geobacter metallireducens* accesses insoluble Fe(III) oxide by chemotaxis. Nature. 2002;416:767–9. 11961561 10.1038/416767a

[bib5] Coker VS, Bell AMT, Pearce CI Time-resolved synchrotron powder X-ray diffraction study of magnetite formation by the Fe(III)-reducing bacterium *Geobacter sulfurreducens*. Am Mineral. 2008;93:540–7.

[bib6] Coker VS, Gault AG, Pearce CI XAS and XMCD evidence for species-dependent partitioning of arsenic during microbial reduction of ferrihydrite to magnetite. Environ Sci Technol. 2006;40:7745–50. 17256522 10.1021/es060990+

[bib7] Cornell RM, Schwertmann U The Iron Oxides: Structure, Properties, Reactions, Occurences and Uses. 2nd edn. London, England: Wiley-VCH, 2006.

[bib8] Cutting RS, Coker VS, Fellowes JW Mineralogical and morphological constraints on the reduction of Fe(III) minerals by *Geobacter sulfurreducens*. Geochim Cosmochim Acta. 2009;73:4004–22.

[bib9] Dang Y, Walker DJF, Vautour KE Arsenic detoxification by *Geobacter* species. Appl Environ Microbiol. 2017;83:e02689–16. 27940542 10.1128/AEM.02689-16 PMC5288829

[bib10] Edwards MJ, White GF, Norman M Redox linked flavin sites in extracellular decaheme proteins involved in microbe-mineral electron transfer. Sci Rep. 2015;5:11677. 26126857 10.1038/srep11677 PMC4486940

[bib11] Fredrickson JK, Romine MF, Beliaev AS Towards environmental systems biology of *Shewanella*. Nat Rev Microbiol. 2008;6:592–603. 18604222 10.1038/nrmicro1947

[bib12] Gault AG, Jana J, Chakraborty S Preservation strategies for inorganic arsenic species in high iron, low-Eh groundwater from West Bengal, India. Anal Bioanal Chem. 2005;381:347–53. 15558247 10.1007/s00216-004-2861-1

[bib13] Gieg LM, Fowler SJ, Berdugo-Clavijo C Syntrophic biodegradation of hydrocarbon contaminants. Curr Opin Biotechnol. 2014;27:21–9. 24863893 10.1016/j.copbio.2013.09.002

[bib14] Giloteaux L, Holmes DE, Williams KH Characterization and transcription of arsenic respiration and resistance genes during in situ uranium bioremediation. ISME J. 2013;7:370–83. 23038171 10.1038/ismej.2012.109 PMC3554400

[bib15] Gnanaprakasam ET, Lloyd JR, Boothman C Microbial community structure and arsenic biogeochemistry in two arsenic-impacted aquifers in Bangladesh. MBio. 2017;8:e01326–17. 29184025 10.1128/mBio.01326-17 PMC5705915

[bib16] Green-Saxena A, Dekas AE, Dalleska NF Nitrate-based niche differentiation by distinct sulfate-reducing bacteria involved in the anaerobic oxidation of methane. ISME J. 2014;8:150–63. 24008326 10.1038/ismej.2013.147 PMC3869021

[bib17] Hansel CM, Benner SG, Neiss J Secondary mineralization pathways induced by dissimilatory iron reduction of ferrihydrite under advective flow. Geochim Cosmochim Acta. 2003;67:2977–92.

[bib18] Harris HW, El-Naggar MY, Bretschger O Electrokinesis is a microbial behavior that requires extracellular electron transport. Proc Natl Acad Sci USA. 2010;107:326–31. 20018675 10.1073/pnas.0907468107 PMC2806741

[bib19] Harris HW, El-Naggar MY, Nealson KH Shewanella oneidensis MR-1 chemotaxis proteins and electron-transport chain components essential for congregation near insoluble electron acceptors. Biochem Soc Trans. 2012;40:1167–77. 23176449 10.1042/BST20120232

[bib20] Herrmann AM, Clode PL, Fletcher IR A novel method for the study of the biophysical interface in soils using nano-scale secondary ion mass spectrometry. Rapid Commun Mass Spectrom. 2007a;21:29–34. 17131465 10.1002/rcm.2811

[bib21] Herrmann AM, Ritz K, Nunan N Nano-scale secondary ion mass spectrometry — A new analytical tool in biogeochemistry and soil ecology: a review article. Soil Biol Biochem. 2007b;39:1835–50.

[bib22] Islam FS, Gault AG, Boothman C Role of metal-reducing bacteria in arsenic release from Bengal delta sediments. Nature. 2004a;430:68–71. 15229598 10.1038/nature02638

[bib23] Islam FS, Pederick RL, Gault AG Interactions between the Fe(III)-reducing bacterium *Geobacter sulfurreducens* and arsenate, and capture of the metalloid by biogenic Fe(II). Appl Environ Microbiol. 2005;71:8642–8. 16332858 10.1128/AEM.71.12.8642-8648.2005 PMC1317334

[bib24] Islam FS, Pederick RL, Polya DA Reduction of Fe(III) by *Geobacter sulfurreducens* and the capture of arsenic by biogenic Fe(II) minerals. Geochim Cosmochim Acta. 2004b;68:A518.

[bib25] Jiang S, Lee J-H, Kim D Differential arsenic mobilization from As-bearing ferrihydrite by iron-respiring Shewanella strains with different arsenic-reducing activities. Environ Sci Technol. 2013;47:8616–23. 23802758 10.1021/es400534z

[bib26] Jiang S, Lee J-H, Kim M-G Biogenic formation of As-S nanotubes by diverse *Shewanella* strains. Appl Environ Microbiol. 2009;75:6896–9. 19717628 10.1128/AEM.00450-09 PMC2772418

[bib27] Jiang X, Hu J, Fitzgerald LA Probing electron transfer mechanisms in *Shewanella oneidensis* MR-1 using a nanoelectrode platform and single-cell imaging. Proc Natl Acad Sci USA. 2010;107:16806–10. 20837546 10.1073/pnas.1011699107 PMC2947899

[bib28] Kerkhof LJ, Williams KH, Long PE Phase preference by active, acetate-utilizing bacteria at the Rifle, CO Integrated Field Research Challenge site. Environ Sci Technol. 2011;45:1250–6. 21226528 10.1021/es102893r

[bib29] Kotloski NJ, Gralnick JA Flavin electron shuttles dominate extracellular electron transfer by *Shewanella oneidensis*. MBio. 2013;4:e00553–12. 23322638 10.1128/mBio.00553-12 PMC3551548

[bib30] Leang C, Coppi M V, Lovley DR OmcB, a *c*-type polyheme cytochrome, involved in Fe(III) reduction in *Geobacter sulfurreducens*. J Bacteriol. 2003;185:2096–103. 12644478 10.1128/JB.185.7.2096-2103.2003 PMC151516

[bib31] Leang C, Qian X, Mester T Alignment of the *c*-type cytochrome OmcS along pili of *Geobacter sulfurreducens*. Appl Environ Microbiol. 2010;76:4080–4. 20400557 10.1128/AEM.00023-10 PMC2893476

[bib32] Levar CE, Hoffman CL, Dunshee AJ Redox potential as a master variable controlling pathways of metal reduction by *Geobacter sulfurreducens*. ISME J. 2016;11:741–52. 10.1038/ismej.2016.146 PMC5322298 28045456

[bib33] Lies DP, Hernandez ME, Kappler A Shewanella oneidensis MR-1 uses overlapping pathways for iron reduction at a distance and by direct contact under conditions relevant for biofilms. Appl Environ Microbiol. 2005;71:4414–26. 16085832 10.1128/AEM.71.8.4414-4426.2005 PMC1183279

[bib34] Liu Y, Fredrickson JK, Zachara JM Direct involvement of *ombB, omaB*, and *omcB* genes in extracellular reduction of Fe(III) by *Geobacter sulfurreducens* PCA. Front Microbiol. 2015;6:1075. 26483786 10.3389/fmicb.2015.01075 PMC4589669

[bib35] Lloyd JR, Blunt-Harris EL, Lovley DR The periplasmic 9.6-kilodalton *c*-type cytochrome of *Geobacter sulfurreducens* is not an electron shuttle to Fe(III). J Bacteriol. 1999;181:7647–9. 10601229 10.1128/jb.181.24.7647-7649.1999 PMC94229

[bib36] Lloyd JR, Gault A, Héry M Microbial transformations of arsenic in the subsurface. In: Stolz J, Oremland R (eds). Microbial Metal and Metalloid Metabolism. Washington, DC.: ASM Press, 2011, 77–90.

[bib37] Lloyd JR, Sole VA, Van Praagh CVG Direct and Fe(II)-mediated reduction of technetium by Fe(III)-reducing bacteria. Appl Environ Microbiol. 2000;66:3743–9. 10966385 10.1128/aem.66.9.3743-3749.2000 PMC92215

[bib38] Lovley DR Long-range electron transport to Fe(III) oxide via pili with metallic-like conductivity. Biochem Soc Trans. 2012;40:1186–90. 23176452 10.1042/BST20120131

[bib39] Lovley DR Electrically conductive pili: biological function and potential applications in electronics. Curr Opin Electrochem. 2017;4:190–8.

[bib40] Lovley DR, Holmes DE, Nevin KP Dissimilatory Fe(III) and Mn(IV) reduction. In: Poole RK (ed). Advances in Microbial Physiology. Academic Press, Cambridge, Massachusetts, USA, 2004, 49:219–86. 10.1016/S0065-2911(04)49005-5 15518832

[bib41] Lovley DR, Phillips EJ Organic matter mineralization with reduction of ferric iron in anaerobic sediments. Appl Environ Microbiol. 1986;51:683–9. 16347032 10.1128/aem.51.4.683-689.1986 PMC238947

[bib42] Lovley DR, Phillips EJP Novel mode of microbial energy metabolism: organic carbon oxidation coupled to dissimilatory reduction of iron or manganese. Appl Environ Microbiol. 1988;54:1472–80. 16347658 10.1128/aem.54.6.1472-1480.1988 PMC202682

[bib43] Lovley DR, Phillips EJP, Gorby YA Microbial reduction of uranium. Nature. 1991;350:413–6.

[bib44] Lovley DR, Phillips EJP, Lonergan DJ Hydrogen and formate oxidation coupled to dissimilatory reduction of iron or manganese by *Alteromonas putrefaciens*. Appl Environ Microbiol. 1989;55:700–6. 16347876 10.1128/aem.55.3.700-706.1989 PMC184183

[bib45] Lovley DR, Stolz JF, Nord GL Anaerobic production of magnetite by a dissimilatory iron-reducing microorganism. Nature. 1987;330:252–4.

[bib46] Malvankar NS, Tuominen MT, Lovley DR Lack of cytochrome involvement in long-range electron transport through conductive biofilms and nanowires of *Geobacter sulfurreducens*. Energy Environ Sci. 2012;5:8651–9.

[bib47] Malvankar NS, Vargas M, Nevin KP Tunable metallic-like conductivity in microbial nanowire networks. Nat Nanotechnol. 2011;6:573–9. 21822253 10.1038/nnano.2011.119

[bib48] Marsili E, Baron DB, Shikhare ID *Shewanella* secretes flavins that mediate extracellular electron transfer. Proc Natl Acad Sci. 2008;105:3968–73. 18316736 10.1073/pnas.0710525105 PMC2268775

[bib49] Mehta T, Coppi M V, Childers SE Outer membrane *c*-type cytochromes required for Fe(III) and Mn(IV) oxide reduction in *Geobacter sulfurreducens*. Appl Environ Microbiol. 2005;71:8634–41. 16332857 10.1128/AEM.71.12.8634-8641.2005 PMC1317342

[bib50] Miot J, Remusat L, Duprat E Fe biomineralization mirrors individual metabolic activity in a nitrate-dependent Fe(II)-oxidizer. Front Microbiol. 2015;6:879. 26441847 10.3389/fmicb.2015.00879 PMC4562303

[bib51] Muehe EM, Morin G, Scheer L Arsenic(V) incorporation in vivianite during microbial reduction of arsenic(V)-bearing biogenic Fe(III) (oxyhydr)oxides. Environ Sci Technol. 2016;50:2281–91. 26828118 10.1021/acs.est.5b04625

[bib52] Musat N, Halm H, Winterholler B A single-cell view on the ecophysiology of anaerobic phototrophic bacteria. Proc Natl Acad Sci. 2008;105:17861–6. 19004766 10.1073/pnas.0809329105 PMC2582579

[bib53] Musat N, Stryhanyuk H, Bombach P The effect of FISH and CARD-FISH on the isotopic composition of ^13^C- and ^15^N-labeled *Pseudomonas putida* cells measured by NanoSIMS. Syst Appl Microbiol. 2014;37:267–76. 24702905 10.1016/j.syapm.2014.02.002

[bib54] Myers CR, Nealson KH Bacterial manganese reduction and growth with manganese oxide as the sole electron acceptor. Science. 1988;240:1319–21. 17815852 10.1126/science.240.4857.1319

[bib55] Nevin KP, Lovley DR Lack of production of electron-shuttling compounds or solubilization of Fe(III) during reduction of insoluble Fe(III) oxide by Geobacter metallireducens. Appl Environ Microbiol. 2000;66:2248–51. 10788411 10.1128/aem.66.5.2248-2251.2000 PMC101484

[bib56] Newsome L, Morris K, Lloyd JR The biogeochemistry and bioremediation of uranium and other priority radionuclides. Chem Geol. 2014;363:164–84.

[bib57] Okamoto A, Hashimoto K, Nakamura R Long-range electron conduction of *Shewanella* biofilms mediated by outer membrane *c*-type cytochromes. Bioelectrochemistry. 2012;85:61–5. 22222436 10.1016/j.bioelechem.2011.12.003

[bib58] Okamoto A, Kalathil S, Deng X Cell-secreted flavins bound to membrane cytochromes dictate electron transfer reactions to surfaces with diverse charge and pH. Sci Rep. 2014;4:141–7. 10.1038/srep05628 PMC4092373 25012073

[bib59] Pirbadian S, Barchinger SE, Leung KM Shewanella oneidensis MR-1 nanowires are outer membrane and periplasmic extensions of the extracellular electron transport components. Proc Natl Acad Sci USA. 2014;111:12883–8. 25143589 10.1073/pnas.1410551111 PMC4156777

[bib60] Ploug H, Musat N, Adam B Carbon and nitrogen fluxes associated with the cyanobacterium *Aphanizomenon* sp. in the Baltic Sea. ISME J. 2010;4:1215–23. 20428225 10.1038/ismej.2010.53

[bib61] Reguera G, McCarthy KD, Mehta T Extracellular electron transfer via microbial nanowires. Nature. 2005;435:1098–101. 15973408 10.1038/nature03661

[bib62] Revesz E, Fortin D, Paktunc D Reductive dissolution of arsenical ferrihydrite by bacteria. Appl Geochem. 2016;66:129–39.

[bib63] Saltikov CW, Cifuentes A, Venkateswaran K The *ars* detoxification system is advantageous but not required for As(V) respiration by the genetically tractable *Shewanella* species strain ANA-3. Appl Environ Microbiol. 2003;69:2800–9. 12732551 10.1128/AEM.69.5.2800-2809.2003 PMC154534

[bib64] Saltikov CW, Newman DK Genetic identification of a respiratory arsenate reductase. Proc Natl Acad Sci. 2003;100:10983–8. 12939408 10.1073/pnas.1834303100 PMC196913

[bib65] Saltikov CW, Wildman RA, Newman DK Expression dynamics of arsenic respiration and detoxification in *Shewanella* sp. strain ANA-3. J Bacteriol. 2005;187:7390–6. 16237022 10.1128/JB.187.21.7390-7396.2005 PMC1272973

[bib66] Schneider C a, Rasband WS, Eliceiri KW NIH Image to ImageJ: 25 years of image analysis. Nat Methods. 2012;9:671–5. 22930834 10.1038/nmeth.2089 PMC5554542

[bib67] Schurig C, Mueller CW, Höschen C Methods for visualising active microbial benzene degraders in in situ microcosms. Appl Microbiol Biotechnol. 2015;99:957–68. 25194840 10.1007/s00253-014-6037-4

[bib68] Schurig C, Smittenberg RH, Berger J Microbial cell-envelope fragments and the formation of soil organic matter: a case study from a glacier forefield. Biogeochemistry. 2013;113:595–612.

[bib69] Schwertmann U, Cornell RM Iron Oxides in the Laboratory. 2nd edn. Berlin, Germany: Wiley-VCH Verlag GmbH, 2000.

[bib70] Seeliger S, Cord-Ruwisch R, Schink B A periplasmic and extracellular *c*-type cytochrome of *Geobacter sulfurreducens* acts as a ferric iron reductase and as an electron carrier to other acceptors or to partner bacteria. J Bacteriol. 1998;180:3686–91. 9658015 10.1128/jb.180.14.3686-3691.1998 PMC107340

[bib71] Singh R, Singh S, Parihar P Arsenic contamination, consequences and remediation techniques: a review. Ecotoxicol Environ Saf. 2015;112:247–70. 25463877 10.1016/j.ecoenv.2014.10.009

[bib72] Smedley PL, Kinniburgh DG A review of the source, behaviour and distribution of arsenic in natural waters. Appl Geochem. 2002;17:517–68.

[bib73] Smith JA, Tremblay P-L, Shrestha PM Going wireless: Fe(III) oxide reduction without pili by *Geobacter sulfurreducens* Strain JS-1. Appl Environ Microbiol. 2014;80:4331–40. 24814783 10.1128/AEM.01122-14 PMC4068678

[bib74] Straub KL, Schink B Evaluation of electron-shuttling compounds in microbial ferric iron reduction. FEMS Microbiol Lett. 2003;220:229–33. 12670685 10.1016/S0378-1097(03)00130-7

[bib75] Subramanian P, Pirbadian S, El-Naggar MY Ultrastructure of *Shewanella oneidensis* MR-1 nanowires revealed by electron cryotomography. Proc Natl Acad Sci USA. 2018, 115(14):E3246–E3255. 29555764 10.1073/pnas.1718810115 PMC5889646

[bib76] Vogel C, Mueller CW, Höschen C Submicron structures provide preferential spots for carbon and nitrogen sequestration in soils. Nat Commun. 2014;5:2947. 24399306 10.1038/ncomms3947 PMC3896754

[bib77] Wang YH, Morin G, Ona-Nguema G Arsenic(III) and arsenic(V) speciation during transformation of lepidocrocite to magnetite. Environ Sci Technol. 2014;48:14282–90. 25425339 10.1021/es5033629

[bib78] Watts MP, Coker VS, Parry SA Biogenic nano-magnetite and nano-zero valent iron treatment of alkaline Cr(VI) leachate and chromite ore processing residue. Appl Geochem. 2015;54:27–42. 26109747 10.1016/j.apgeochem.2014.12.001 PMC4461148

[bib79] White GF, Edwards MJ, Gomez-Perez L Mechanisms of bacterial extracellular electron exchange. In: Poole RK (ed). Advances in Microbial Physiology. Academic Press, Cambridge, Massachusetts, USA, 2016, 68:87–138. 10.1016/bs.ampbs.2016.02.002 27134022

[bib80] Wilkins MJ, VerBerkmoes NC, Williams KH Proteogenomic monitoring of *Geobacter* physiology during stimulated uranium bioremediation. Appl Environ Microbiol. 2009;75:6591–9. 19717633 10.1128/AEM.01064-09 PMC2765142

[bib81] Williams KH, Long PE, Davis JA Acetate availability and its influence on sustainable bioremediation of uranium-contaminated groundwater. Geomicrobiol J. 2011;28:519–39.

[bib82] Woebken D, Burow LC, Prufert-Bebout L Identification of a novel cyanobacterial group as active diazotrophs in a coastal microbial mat using NanoSIMS analysis. ISME J. 2012;6:1427–39. 22237543 10.1038/ismej.2011.200 PMC3379636

[bib83] Wu Y, Ke X, Hernández M Autotrophic growth of bacterial and archaeal ammonia oxidizers in freshwater sediment microcosms incubated at different temperatures. Appl Environ Microbiol. 2013;79:3076–84. 23455342 10.1128/AEM.00061-13 PMC3623126

[bib84] Xu S, Jangir Y, El-Naggar MY Disentangling the roles of free and cytochrome-bound flavins in extracellular electron transport from *Shewanella oneidensis* MR-1. Electrochim Acta. 2016;198:49–55.

