# Supplementary material for: NanoSIMS imaging of extracellular electron transport processes during microbial iron(III) reduction
Source: FEMS Microbiol Ecol. 2018 Jun 6;94(8):fiy104. doi: 10.1093/femsec/fiy104 (PMC6041951; doi:10.1093/femsec/fiy104)
Supplement: Supplementary Data [file fiy104_supplemental_files.zip › supporting information.docx]

**SUPPORTING TABLES**

Table S1. Correction factors used to normalise for total image dose

|  |  | **Number of scans performed** | **Total dose implanted during imaging (Cs atoms cm^-2^)** | **Dose correction factor applied to dataset *** |
| --- | --- | --- | --- | --- |
| *G.sulf* | Area 1 | 10 | 3.94E+15 | 3.94 |
| *G.sulf* | Area 2 | 160 | 4.03E+16 | 40.32 |
| *G.sulf* | Area 3 | 10 | 1.19E+15 | 1.19 |
| *G.sulf* | Area 4 | 15 | 1.37E+15 | 1.37 |
| *G.sulf* | Area 5 | 85 | 1.83E+16 | 18.26 |
| *G.sulf* | Area 6 | 150 | 1.79E+16 | 17.88 |
| *G.sulf* | Area 7 | 150 | 1.79E+16 | 17.88 |
| *G.sulf* | Area 8 | 90 | 5.45E+15 | 5.45 |
| *G.sulf* | Area 9 | 150 | 1.15E+16 | 11.51 |
| *S.*ANA3 | Area 1 | 20 | 6.11E+15 | 6.11 |
| *S.*ANA3 | Area 2 | 17 | 2.55E+15 | 2.55 |
| *S.*ANA3 | Area 3 | 10 | 1.50E+15 | 1.50 |

*The dose correction factor was obtained by dividing the total dose implanted by 1 x 10^15^

**SUPPORTING FIGURES**


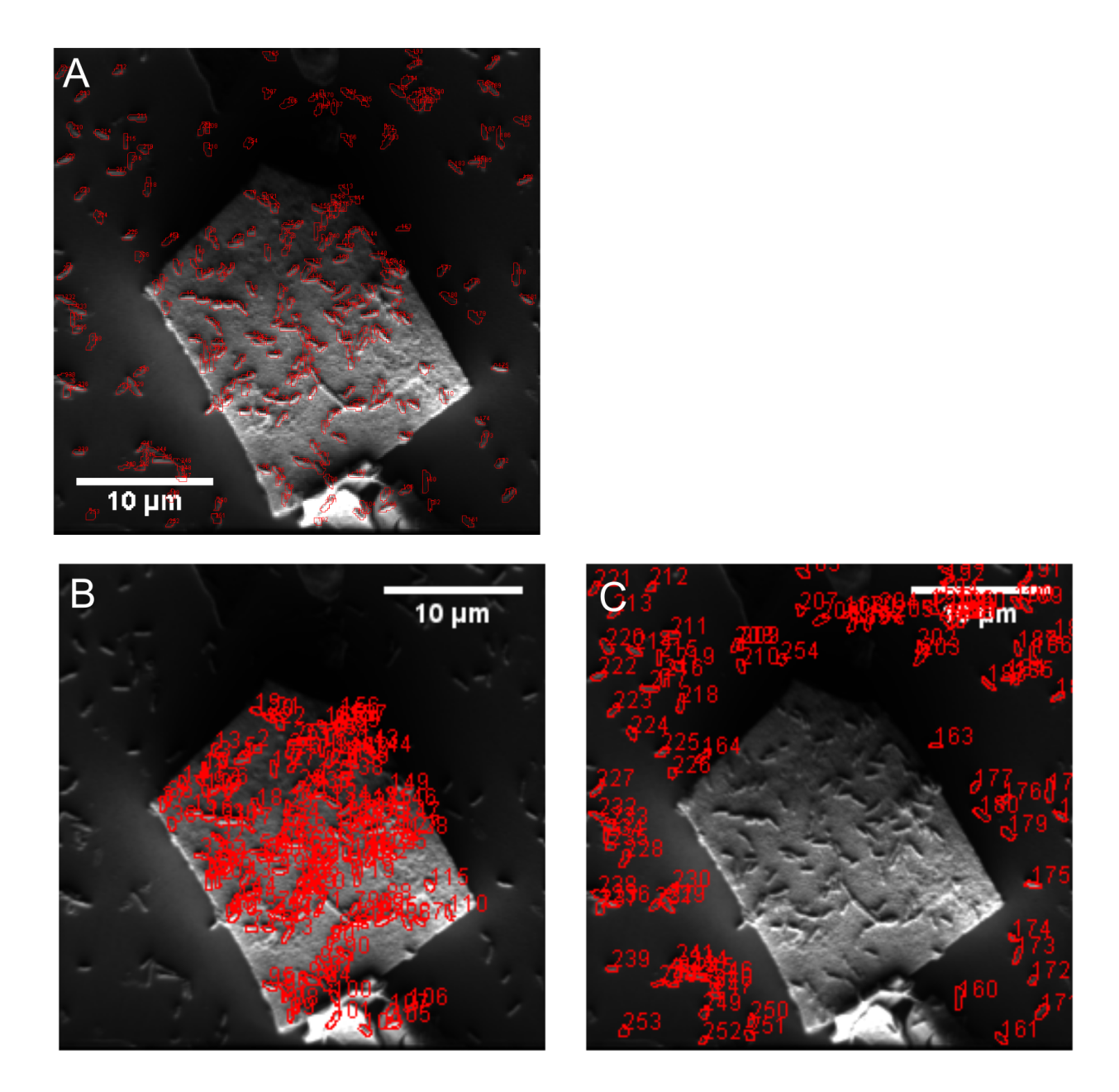


Figure S1. (a) Example showing all cell Regions of Interest (ROIs) defined for Area 6 of the *Geobacter sulfurreducens* experiment. (b) ROIs for cells on the mineral surface. (c) ROIs for cells on the wafer background.

***
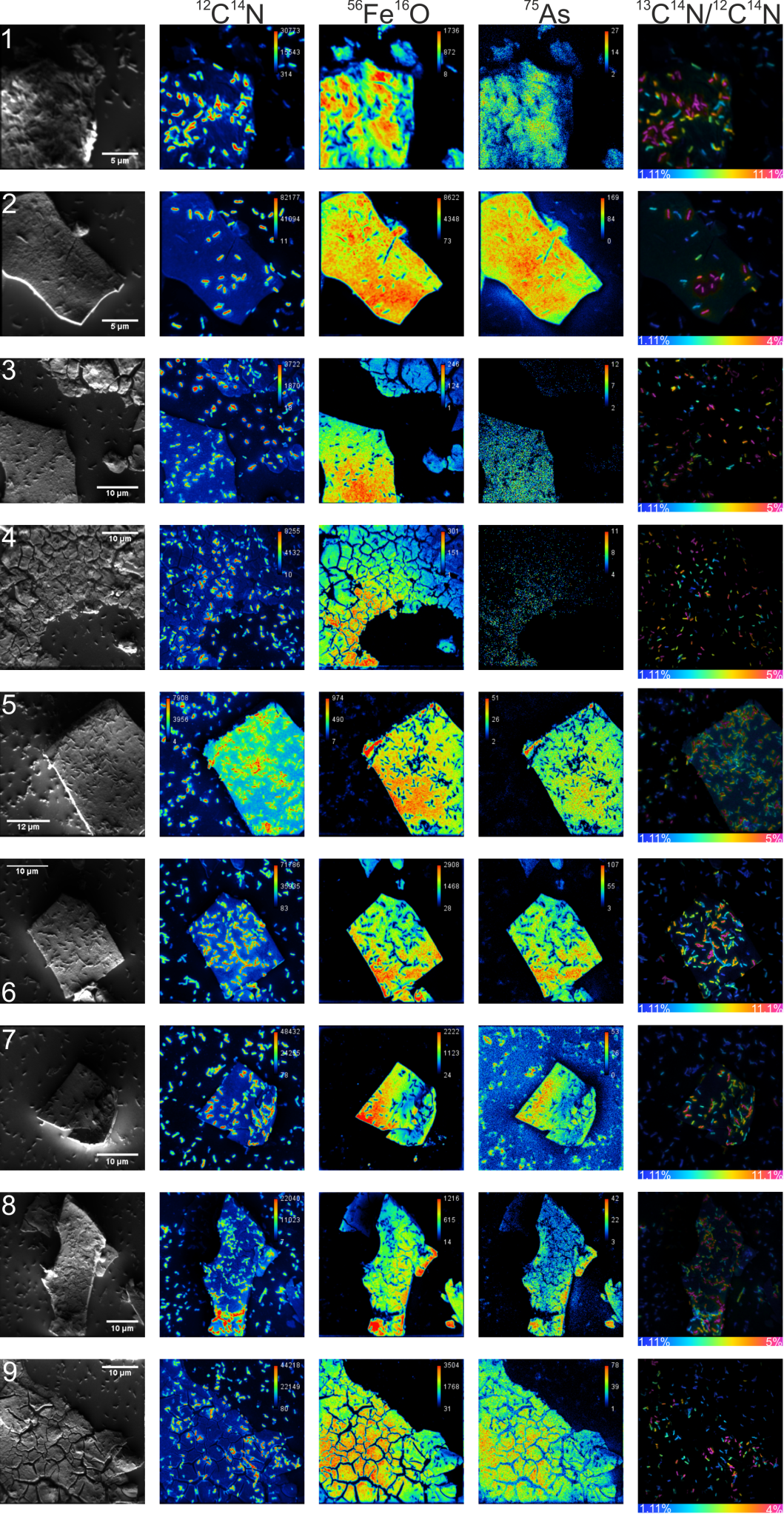
***

Figure S2. Images for the nine areas of the *Geobacter sulfurreducens* sample that were analysed with NanoSIMS. The first column shows secondary electron intensity distribution maps. ^12^C^14^N shows where biomass was present. ^56^Fe^16^O shows the iron oxide mineral. The ^75^As images are likely to show some contribution from the ^56^Fe^19^F interference. ^13^C enrichment was calculated as a ratio of ^13^C^14^N to ^12^C^14^N.


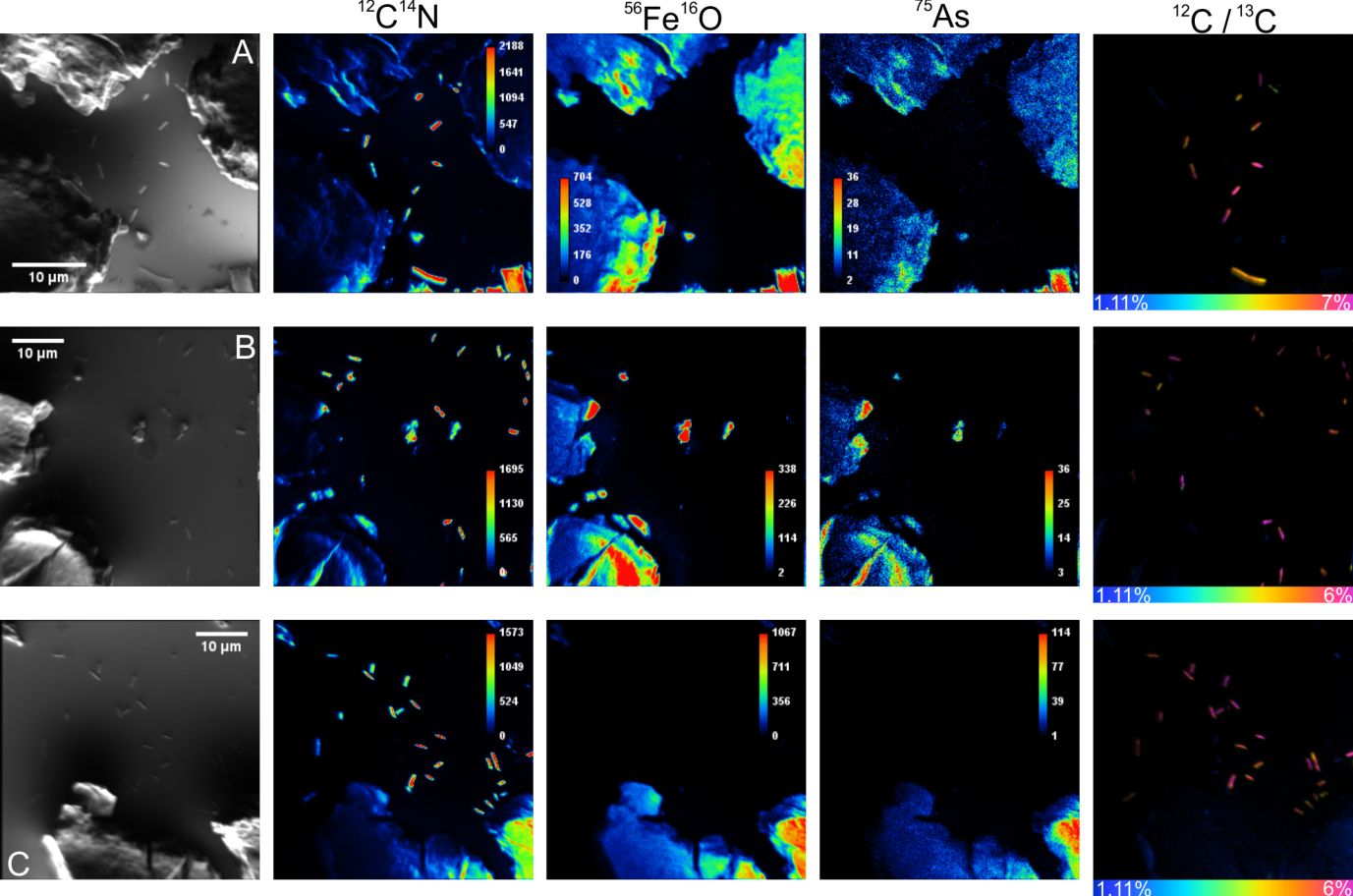


Figure S3. Images for the three areas of the *S*. ANA-3 sample that were analysed with NanoSIMS. The first column shows secondary electron intensity distribution maps. ^12^C^14^N shows where biomass was present. ^56^Fe^16^O shows the iron oxide mineral. The ^75^As images show co-localization with the mineral and the ^56^Fe^16^O signal. ^13^C enrichment was calculated as a ratio of ^13^C to ^12^C.





Figure S4. Box and whisker plots of ^75^As counts as a percentage of ^56^Fe^16^O counts illustrated for the entire *Geobacter sulfurreducens* dataset (same data as Figure 7, here far left grey/pink) and for the nine areas of the sample analysed (black/red). Black/grey boxes show data for regions of interest (ROIs) on the mineral surface, red/pink boxes are ROIs of the cells located on the mineral. N refers to the number of ROIs. Results clearly demonstrate that in 8 of the 9 areas analysed there was a higher proportion of ^75^As relative to ^56^Fe^16^O in the local environment of the cells compared to the un-colonised mineral surface (P < 0.001, Tukey-Kramer test).

**
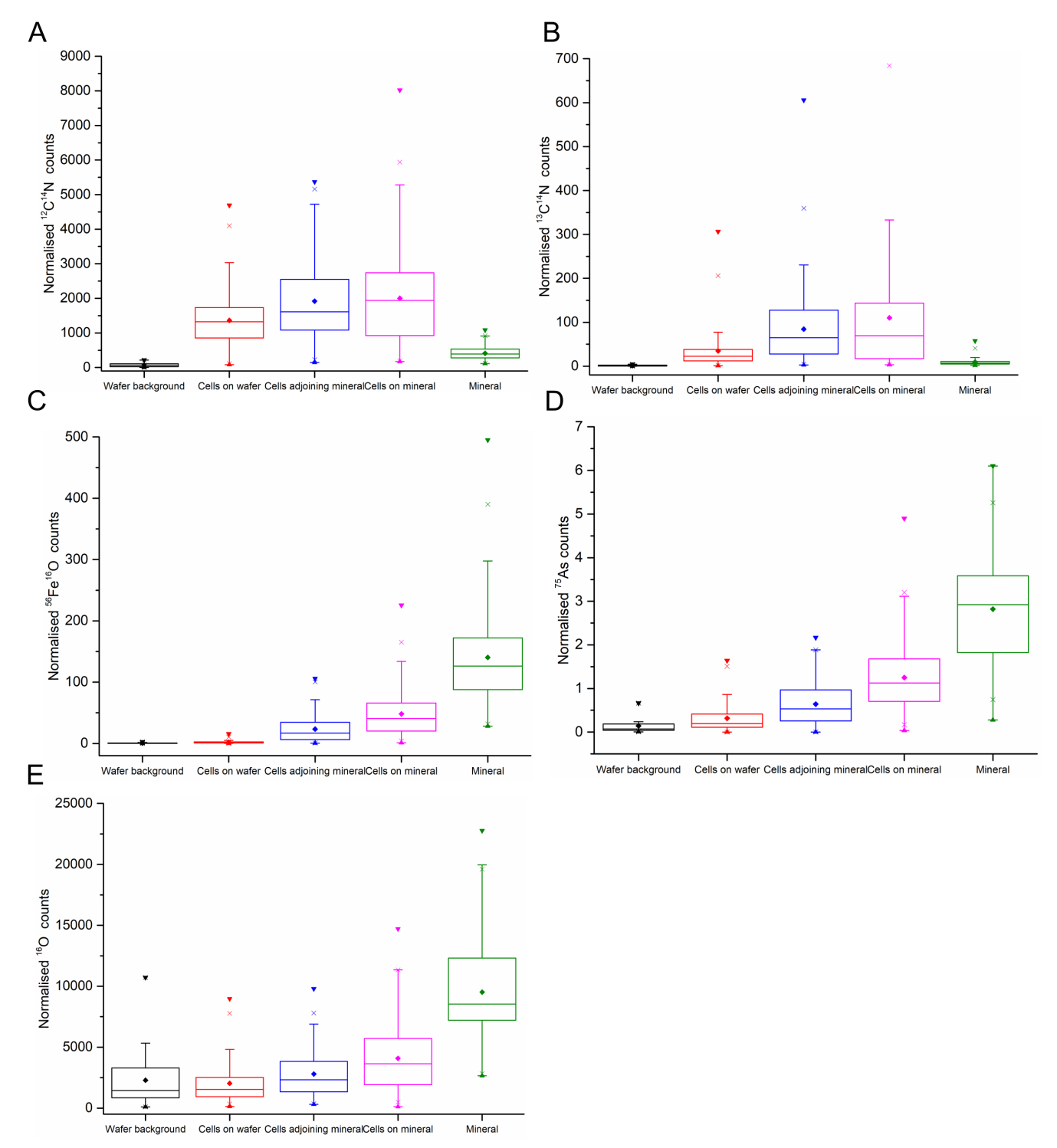
**

Figure S5. Normalised count data for the entire *Geobacter sulfurreducens* dataset. (a) ^12^C^14^N. (b) ^13^C^14^N. (c) ^56^Fe^16^O. (d) ^75^As. (e) ^16^O.
